# Collision cross‐section analysis of self‐assembled metallomacrocycle isomers and isobars via ion mobility mass spectrometry

**DOI:** 10.1002/rcm.8717

**Published:** 2020-02-08

**Authors:** Kevin J. Endres, Kevin Barthelmes, Andreas Winter, Robert Antolovich, Ulrich S. Schubert, Chrys Wesdemiotis

**Affiliations:** ^1^ Department of Polymer Science University of Akron Akron OH USA; ^2^ Laboratory of Organic and Macromolecular Chemistry (IOMC) Friedrich Schiller University Jena Humboldtstr. 10 Jena Germany; ^3^ Department of Materials and Applied Chemistry Nihon University 1‐8‐14 Kanda Surugadai Chiyoda‐ku Tokyo Japan; ^4^ Jena Center for Soft Matter (JCSM) Friedrich Schiller Universität Jena Philosophenweg 7 Jena Germany; ^5^ Department of Chemistry University of Akron Akron OH USA

## Abstract

**Rationale:**

Coordinatively driven self‐assembly of transition metal ions and bidentate ligands gives rise to organometallic complexes that usually contain superimposed isobars, isomers, and conformers. In this study, the double dispersion ability of ion mobility mass spectrometry (IM‐MS) was used to provide a comprehensive structural characterization of the self‐assembled supramolecular complexes by their mass and charge, revealed by the MS event, and their shape and collision cross‐section (Ω), revealed by the IM event.

**Methods:**

Self‐assembled complexes were synthesized by reacting a bis(terpyridine) ligand exhibiting a 60^o^ dihedral angle between the two ligating terpyridine sites (**T**) with divalent Zn, Ni, Cd, or Fe. The products were isolated as (Metal^2+^[**T**])_*n*_ (PF_6_)_2*n*_ salts and analyzed using IM‐MS after electrospray ionization (ESI) which produced several charge states from each *n*‐mer, depending on the number of PF_6_ˉ anions lost upon ESI. Experimental Ω data, derived using IM‐MS, and computational Ω predictions were used to elucidate the size and architecture of the complexes.

**Results:**

Only macrocyclic dimers, trimers, and tetramers were observed with Cd^2+^, whereas Zn^2+^ formed the same plus hexameric complexes. These two metals led to the simplest product distributions and no linear isomers. In sharp contrast, Ni^2+^ and Fe^2+^ formed all possible ring sizes from dimer to hexamer as well as various linear isomers. The experimental and theoretical Ω data indicated rather planar macrocyclic geometries for the dimers and trimers, twisted 3D architectures for the larger rings, and substantially larger sizes with spiral conformation for the linear congeners. Adding PF_6_ˉ to the same complex was found to mainly cause size contraction due to new stabilizing anion–cation interactions.

**Conclusions:**

Complete structural identification could be accomplished using ESI‐IM‐MS. Our results affirm that self‐assembly with Cd^2+^ and Zn^2+^ proceeds through reversible equilibria that generate the thermodynamically most stable structures, encompassing exclusively macrocyclic architectures that readily accommodate the 60^o^ ligand used. In contrast, complexation with Ni^2+^ and Fe^2+^, which form stronger coordinative bonds, proceeds through kinetic control, leading to more complex mixtures and kinetically trapped less stable architectures, such as macrocyclic pentamers and linear isomers.

## 1 INTRODUCTION

Ion mobility mass spectrometry (IM‐MS) is increasingly used to gain detailed insight into the 3D structures of macromolecules and macromolecular complexes of both biological and synthetic nature.^1^, ^2^ When coupled with molecular simulations and modeling, IM‐MS can provide conclusive evidence about the spatial arrangement and conformation of proteins,^3^, ^4^, ^5^ glycans,^6^ and lipids,^7^ as well as the topology and architecture of synthetic polymers^8^, ^9^, ^10^, ^11^, ^12^, ^13^ and bioconjugates.^13^, ^14^, ^15^

Over the past years, significant progress has been made in the development of simple, one‐step, quantitative methods for the conversion of polyfunctional monomers with well‐defined geometries into supramolecular constructs with useful physicochemical properties.^16^, ^17^, ^18^, ^19^, ^20^ Terpyridine (tpy)‐based coordinative chemistry with various metal^2+^ ions, in particular, has enabled the synthesis of a vast array of macrocyclic and multicyclic compounds carrying the <tpy‐Metal^2+^‐tpy> connectivity and having precise metal/ligand stoichiometries and specific, predesigned 3D geometries, depending on the molecular structure of the tpy ligand.^21^, ^22^, ^23^, ^24^, ^25^, ^26^, ^27^, ^28^, ^29^, ^30^, ^31^ The ligand design, type of metal(s), counterions, reaction temperature, and solvent play an important role in the composition, size, and architecture of the resulting macrocyclic system. IM‐MS has been instrumental in establishing the latter structural features with high sensitivity and specificity, especially when mixtures with isobars and isomers are generated that are difficult to characterize by other analytical techniques.^8^, ^21^, ^22^, ^23^, ^24^, ^25^, ^26^, ^27^, ^28^, ^29^, ^30^, ^31^

An important attribute of IM‐MS that sets it apart from other characterization techniques is its ability to separate isobaric and isomeric ions in the gas phase, which is often necessary for samples formed by coordination‐driven self‐assembly reactions. Electrospray ionization (ESI) is the method of choice for the mass spectrometry (MS) analysis of these samples, as most metal ligands contain highly conjugated UV chromophores that preclude the use of laser‐based ionization. ESI‐MS often creates isobaric stoichiometries with overlapping isotope clusters, such as an *n*‐mer with *x* + charges and a 2*n*‐mer with 2*x* + charges; in addition, each of these compositions may comprise more than one isomer.^8^, ^21^, ^22^, ^23^, ^24^, ^25^, ^26^, ^27^, ^28^, ^29^, ^30^, ^31^ Similar to chromatographic fractionation, the IM dimension of IM‐MS can disperse these ions by allowing them to drift under the influence of an electric field against the stream of a buffer gas. This capability of IM‐MS was recently used to investigate concentration‐driven association–dissociation (fusion–fission) equilibria between isobaric complex ions; IM‐MS permitted the separation of the superimposed ions, thereby enabling measurement of their individual abundances for derivation of the corresponding equilibrium constant.^32^

Several variants of IM‐MS exist, depending on the type and strength of the electric field and the pressure of the buffer gas used in the IM region.^33^, ^34^ In all variants, the time needed for an ion to travel through the IM region is defined as the drift time; it depends on the ion's mass, charge, and collision cross‐section (Ω).^1^, ^2^ Ω is a physical property reflecting ion size and 3D shape; this parameter, combined with the mass‐to‐charge ratio (*m/z*) available through the MS dimension, helps to distinguish isobars, isomers, and conformers of organometallic assemblies, thus providing architecture, topology, and conformational information not available by simple *m/z* measurement.^10^, ^13^, ^35^ In addition, the Ω values of individual architectures can be calculated using molecular modeling simulations^36^ for comparison with the experimental results from IM‐MS drift times, so that 3D structural assignments can be made confidently.^10^, ^22^, ^23^, ^26^, ^27^, ^31^, ^35^

In the present study, ESI‐MS and ESI‐IM‐MS are used to characterize a large set of self‐assembled metallo‐supramolecular species synthesized from a single bis (terpyridine) ligand, designed to favor macrocyclization, and four divalent transition metals (Zn^2+^, Ni^2+^, Cd^2+^, Fe^2+^). The species probed span over *m/z* 750–2500 and have Ω values ranging from 600 to 2200 Å^2^ depending on their charge and self‐assembled architecture. The IM dimension is shown to be essential for discerning the stoichiometries formed and rationalizing the architectural diversity observed among the four metals examined.

## 2 EXPERIMENTAL

### 2.1 Synthesis of metalloorganic macrocycles

Bis (terpyridine) ligand **T** was prepared according to a literature procedure.^37^ All other chemicals were purchased from commercial suppliers and used as received. A 20‐mL microwave vial was charged with the ditopic ligand **T** (31 mg, 0.021 mmol) and CHCl_3_ (15 mL), the vial was capped, and the solution was degassed with nitrogen until 5 mL of the CHCl_3_ was evaporated. Another microwave vial (5‐ mL) was charged with equimolar amounts of the metal salt (ie, FeSO_4_ × 7H_2_O, Cd(NO_3_)_2_ × 4H_2_O, Zn(NO_3_)_2_ × 6H_2_O, or Ni(NO_3_)_2_ × 6H_2_O) and MeOH (6 mL), capped, and degassed with nitrogen until 2 mL of the MeOH was evaporated. The metal salt solution was added (1 mL/min) to the stirring ligand solution using a syringe, and the mixture was heated for 18 hours at 40°C. Subsequently, the reaction mixture was cooled to room temperature (in the case of the Fe^2+^ macrocycles, the precipitate formed was separated, purified, and isolated as the PF_6_ˉ salt). The solution was concentrated to 5 mL, and excess NH_4_PF_6_ and MeCN (10 mL) were added; after stirring for 10 minutes, the solution was added to MeOH (50 mL). The precipitate formed was collected by filtration and washed with water, MeOH, and diethyl ether. The solid was redissolved in acetonitrile (5 mL), and the solution was treated with diethyl ether vapor that diffused over 1 week into the solution, forcing the slow precipitation of the complexes as PF_6_ˉ salts. The isolated products were characterized using nuclear magnetic resonance (NMR) spectroscopy and size‐exclusion chromatography (SEC) as detailed in the supporting information.

### 2.2 IM‐MS experiments

All experiments were performed using a Synapt HDMS quadrupole/time‐of‐flight (QTOF) mass spectrometer (Waters Corp., Milford, MA, USA) equipped with the traveling‐wave version of IM‐MS^38^ and ESI.^22^, ^23^ The IM region was located between the Q and TOF mass analyzers, within a triwave device consisting of three cells in the order trap cell, IM cell, and transfer cell. The trap and transfer cells were pressurized with Ar, and the IM cell with N_2_. The following parameters were used: ESI capillary voltage, 3.2 kV; sample cone voltage, 35 V; extraction cone voltage, 3.2 V; desolvation gas flow rate, 500 L/h (N_2_); trap collision energy (CE), 6 eV; transfer CE, 4 eV; trap gas flow rate, 1.5 mL/min (Ar); IM cell gas flow rate, 22.7 mL/min (N_2_); sample flow rate, 5 μL/min; source temperature, 80°C; desolvation temperature, 150°C; IM traveling‐wave height, 7.5 V; and IM traveling‐wave velocity, 350 m/s. The sprayed solutions were prepared by dissolving the sample in MeCN at 0.05 mg/mL. Data analyses were conducted using the MassLynx 4.1 and DriftScope 2.1 programs provided by Waters.

### 2.3 Experimental collision cross‐sections

The drift times measured by IM‐MS were converted into collision cross‐sections by calibrating the drift time scale with standards of known Ω value.^39^ Ubiquitin ions in charge states 4+ to 13+ served as calibrants,^40^ which were analyzed at the same traveling‐wave velocity (350 m/s), traveling‐wave height (7.5 V), and IM gas flow rate (22.7 mL/min) as the metallomacrocycles. The resulting calibration curve is shown in the supporting information.

### 2.4 Molecular modeling

Energy minimization of the different organometallic assemblies was conducted with the Materials Studio version 7.0 program, using the Anneal and Geometry Optimization tasks in the Forcite module (Accelrys Software Inc., San Diego, CA, USA). The PF_6_ˉ counterions were omitted. An initially energy‐minimized structure was subjected to 50 annealing cycles with initial and mid‐cycle temperatures of 400 and 1500 K, respectively, 20 heating ramps per cycle, one thousand dynamics steps per ramp, and one dynamics step per femtosecond. A constant volume/constant energy (NVE) ensemble was used; the geometry was optimized after each cycle. All geometry optimizations used a universal force field with atom‐based summation and cubic spline truncation for both the electrostatic and the van der Waals parameters. Fifty candidate structures were generated for each complex, and their collision cross‐sections, calculated using the trajectory method in the MOBCAL program,^36^ were averaged to obtain a representative Ω value for this complex.

## 3 RESULTS AND DISCUSSION

### 3.1 Preparation and NMR/SEC characterization of the metallomacrocycles

**SCHEME 1:**
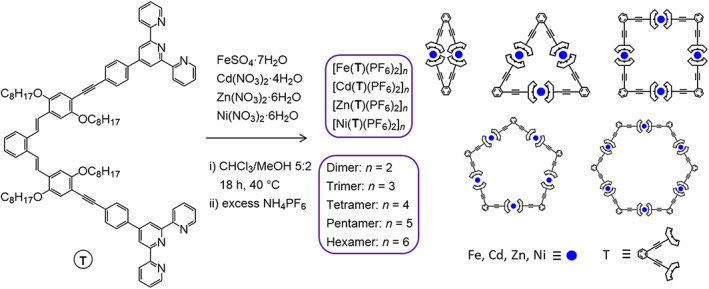
Synthetic route to the metallomacrocycles investigated

Scheme 1 displays the synthetic approach used to prepare self‐assembled metallomacrocycles, ranging from dimers to hexamers (*n* = 2–6), from a single bis (terpyridine)‐based ligand **T** and hydrated divalent metal salts of Fe, Cd, Zn, or Ni. In order to obtain macrocycles in a controlled fashion, high‐dilution and rather weakly binding transition metal ions were used; each of the divalent metal salts and an equimolar amount of **T** were dissolved in chloroform/methanol under slightly elevated temperatures. The complexes were precipitated in a methanolic ammonium hexafluorophosphate solution to generate uniform counter anions. In a second purification step, any remaining uncomplexed ligand **T** was removed by precipitating the macrocycles by slow diffusion of diethyl ether vapor into an acetonitrile solution. Only with the Fe^2+^ salt was a precipitate already formed during the reaction; this material was isolated by filtration and subsequently purified and analyzed independently from the material isolated from the solution. The macrocycles containing Zn^2+^, Cd^2+^, or Fe^2+^ metal centers were analyzed by ^1^H NMR spectroscopy, and the signals could be assigned with the help of 2D NMR spectra (cf. Figures S1–S9, supporting information). The <tpy‐Metal^2+^‐tpy> connectivity involves octahedral coordination of the metal ion by two tpy ligating sites (cf. Scheme 1). Indeed, the ^1^H NMR spectra display the metal‐specific shifts of the terpyridine signals in the respective octahedral complexes (cf. Figure S1, supporting information). All spectra comprise signals of relatively sharp and distinct shapes and show no residual‐free terpyridine signals. This indicates that closed well‐defined structures were formed (such as those depicted in Scheme 1) — in contrast, metallopolymers are reported to exhibit very broad signals and residual non‐coordinated terpyridine signals due to their end groups.^41^ It is noteworthy that the ^1^H NMR spectra of (Fe[**T**])_*n*_ (PF_6_)_*2n*_ from the precipitate and filtrate of the reaction solution look nearly identical (Figure S2, supporting information); nevertheless, the signals in the spectrum of the sample from the filtrate appear broadened, indicating a more uniform macrocyclization in the precipitate. Although the NMR spectra agree well with the formation of macrocycles, they cannot distinguish between differently sized macrocyclic assemblies that may be formed simultaneously. The complexes were therefore analyzed further by SEC to assess the distribution of differently sized metallomacrocycles. Due to the pronounced kinetic lability of the Zn(II)‐ and Cd(II)‐bis(terpyridine) coordinative bonds (cf. Table S1, supporting information), macrocycles with these metal centers fully degrade under SEC analysis. Thus, SEC chromatograms could be obtained only for the Fe(II)‐ and Ni(II)‐bis(terpyridine)‐containing samples that are kinetically stable to withstand SEC conditions.^42^ In the case of the (Fe[**T**])_*n*_ (PF_6_)_2*n*_ precipitate, one sharp peak with the characteristic absorption spectrum of Fe(II)‐bis(terpyridine) complexes is observed (Figure S10, supporting information). In contrast, the chromatogram of (Fe[**T**])_*n*_ (PF_6_)_2*n*_ from the filtrate exhibites a convoluted peak with a component at the same retention time as that from the precipitate, plus a shoulder and fronting at shorter retention times (Figure S11, supporting information). The additional signals, which also correspond to Fe(II)‐bis (terpyridine) species according to their absorption spectra, were tentatively assigned to larger macrocycles. For (Ni[**T**])_*n*_ (PF_6_)_*2n*_, the chromatogram is similar to that of the filtrate‐derived (Fe[**T**])_*n*_ (PF_6_)_2*n*_, strongly suggesting a similar macrocycle distribution (Figure S12, supporting information). Overall, the NMR and SEC results point out that differently sized macrocycles had potentially been formed; however, the nature of these additional products remained ambiguous. For this reason, ESI‐MS and ESI‐IM‐MS were used to gain more insight into the macrocycle architectures formed.

### 3.2 ESI‐MS analysis of the metallomacrocycles

**Figure 1:**
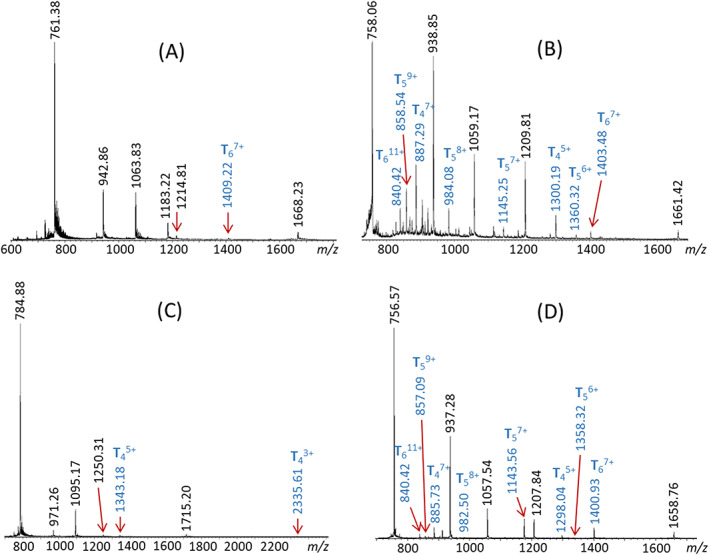
Electrospray ionization mass spectra of A, (Zn[**T**])_*n*_ (PF_6_)_2*n*_, B, (Ni[**T**])_*n*_ (PF_6_)_2*n*_, C, (Cd[**T**])_*n*_ (PF_6_)_2*n*_, and D, (Fe[**T**])_*n*_ (PF_6_)_2*n*_; the labels on top of the peaks indicate the *m/z* value of the most abundant isotope. Charge states with unique *m/z* values (no overlapping compositions) are labeled in blue, and their composition is identified. See Table S2 (supporting information) for the *m/z* values of all possible charge states from these four compounds examined. The (Fe[**T**])_*n*_ (PF_6_)_2*n*_ precipitate was used to acquire the spectrum of the Fe^2+^ complexes

The coordinative bonds formed between bis(terpyridine) ligands, like **T**, and the divalent metals used vary significantly in strength,^41^ causing each ligand–metal combination to yield a distinct distribution of macrocycles comprising *n* Metal^2+^(**T**) repeat units and 2*n* PF_6_ˉ counterions (cf. Scheme 1). ESI of these mixtures gives rise to a series of charge states from each macrocycle, viz. ([Metal^2+^{**T**}]_*n*_ [PF_6_]_2*n*–*x*_)^*x*+^ with *x* designating the number of detached PF_6_ˉ counterions. Furthermore, the stoichiometric regularities in ligand, metal, and counterion content among the different macrocycles result in the prevalence of isobaric ions in the ESI‐MS spectra, as attested in Figure 1 for the complexes obtained from **T** and Zn^2+^, Ni^2+^, Cd^2+^, or Fe^2+^ ions. Overlapping isobaric ions lead to convoluted isotopic distributions, thus making charge and composition elucidation challenging. Most *n*‐mer compositions form overlapping isobaric ions (cf. Table S2, supporting information), but stoichiometries with unique *m/z* values also exist (labeled in blue in Figure 1) and provide direct evidence for the formation of the corresponding macrocycles. The ions detected in the ESI‐MS spectra in Figure 1 verify the formation of hexamers for Zn^2+^, Ni^2+^, and Fe^2+^; pentamers for Ni^2+^ and Fe^2+^; and tetramers for Ni^2+^, Cd^2+^, and Fe^2+^.

Figure 1 clearly shows that the four metal ions investigated yield different mixtures of organometallic assemblies upon reaction with bis(terpyridine) ligand **T**. The spectral complexity increases in the order Figure 1C (Cd^2+^ complexes) < Figure 1A (Zn^2+^ complexes) < Figure 1B (Ni^2+^ complexes) ≈ Figure 1D (Fe^2+^ complexes). The association constants for complexation of divalent transition metals with bis (terpyridine) ligands and the stabilities of the resulting <tpy‐Metal^2+^‐tpy> coordinative bonds increase in a similar order, viz. Cd^2+^ < Zn^2+^ < Ni^2+^ < Fe^2+^ (cf. Table S1, supporting information).^19^, ^41^, ^43^, ^44^ For the metal ions forming weak coordinative bonds (Cd^2+^ and Zn^2+^), complex formation can proceed through reversible equilibria that enable interconversion into rings with thermodynamically more favored sizes, thus leading to relatively few products. On the contrary, the strong coordinative bonds generated with Fe^2+^ and Ni^2+^ ions produce kinetically stable complexes and equilibration is hampered; in these cases, the range of initially formed macrocycles is “trapped” and observed using ESI‐MS (e.g., pentamers).

**Figure 2:**
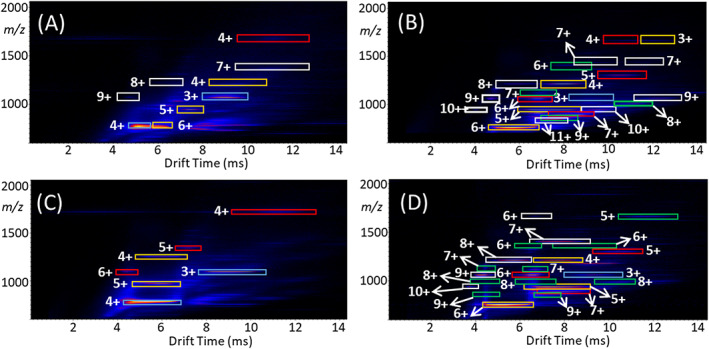
Electrospray ionization ion mobility mass spectrometry (ESI‐IM‐MS) mobilograms of A, (Zn[**T**])_*n*_ (PF_6_)_2*n*_, B, (Ni[**T**])_*n*_ (PF_6_)_2*n*_, C, (Cd[**T**])_*n*_ (PF_6_)_2*n*_, and D, (Fe[**T**])_*n*_ (PF_6_)_2*n*_ complexes. Mobility‐separated bands are encased in colored boxes: blue for dimers, yellow for trimers, red for tetramers, green for pentamers, and white for hexamers. The breadth of structures varies from metal to metal. The charge states are labeled next to each boxed mobility band. Bands of low intensity are not marked to avoid congestion; see Table S3 (supporting information) for a complete list of all ions detected above noise level in these mobilograms

A detailed characterization of the various macrocycle architectures formed during the coordinative self‐assembly of ligand **T** with Zn^2+^, Ni^2+^, Cd^2+^, and Fe^2+^ ions was completed using ESI‐IM‐MS. The added IM dimension helped to separate ions with different shapes and charges overlapping at the same *m/z* value, thereby providing the opportunity to determine the macrocycle sizes produced as well as the presence of any linear architectures. The 2D mobilograms (plots of ion drift time through the IM region vs the respective *m/z value*) of the four systems studied are displayed in Figure 2. They all confirm the existence of superimposed isobars, such as the Zn^2+^‐containing ions with 4+ and 6+ charges at *m/z* 761 (Figure 2A), the Ni^2+^‐containing ions with 4+ and 3+ charges at *m/z* 1661 (Figure 2B), the Cd^2+^‐containing ions with 6+ and 3+ charges at *m/z* 1095 (Figure 2C), or the Fe^2+^‐containing ions with 8+ and 4+ charges at *m/z* 1208 (Figure 2D). The mobilograms also attest the existence of isomers or conformers within the Ni^2+^ and Fe^2+^ complex mixtures, such as the two ions with 9+ charges at *m/z* 1059 in Figure 2B and the three ions with 8+ charges at *m/z* 982 in Figure 2D. This separation results from differences in the collision cross‐sections (Ω) of the overlapping ions (true for isomers and conformers) or from their distinct charges in relationship to their collision cross‐sections (true for isobars). Ions with higher charge and smaller Ω value traverse the IM chamber more quickly (and are detected at shorter drift times in the mobilograms) and vice versa.

**Figure 3:**
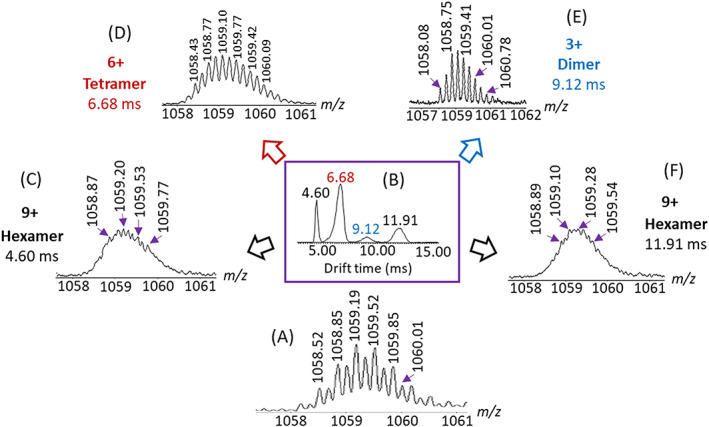
A, Zoomed view of the *m/z* 1058–1061 window in the ESI‐MS spectrum of (Ni[**T**])_*n*_ (PF_6_)_2*n*_ (Figure 1B) encompassing superimposed isobars and isomers, which are separated by B, ESI‐IM‐MS into four components with drift time distributions peaking at 4.60, 6.68, 9.12, and 11.91 ms; these four peaks are shown in band form in the mobilogram of Figure 2B. (C–F) Mass spectra extracted from the four mobility separated peaks; based on the observed isotope spacings and *m/z* values, these peaks are assigned to C, ([Ni{**T**}]_6_[PF_6_]_3_)^9+^ (cyclic hexamer with 9+ charges), D, ([Ni{**T**}]_4_[PF_6_]_2_)^6+^ (cyclic tetramer with 6+ charges), E, ([Ni{**T**}]_2_[PF_6_])^3+^ (cyclic dimer with 3+ charges), and F, ([Ni{**T**}]_6_[PF_6_]_3_)^9+^ (linear hexamer with 9+ charges). Linear and cyclic ([Ni{**T**}]_6_[PF_6_]_3_)^9+^ are isomers, and the other complexes are isobars

The composition (ie, *n*‐mer size) and charge of the ions in each band of the mobilograms are identified from the *m/z* value and isotope pattern observed in the extracted mass spectra, as documented in Figure 3 for the Ni^2+^‐containing bands at *m/z* 1059 (transmitted window *m/z* 1058–1061). The distance between adjacent isotopes is 1/*z*, thus revealing the charge. Multiplying *z* by *m/z* renders the mass and, therefore, the *n*‐mer size, as indicated in Figure 3, which shows the mass spectra extracted for the four IM‐MS bands encased at *m/z* 1059 in Figure 2B.

The charge of the different ions detected is determined by the number of PF_6_ˉ counterions lost during ESI (vide supra). All ESI‐IM‐MS mobilograms include single bands for “naked” (ie, counterion‐free) *n*‐mers, which give rise to the most abundant ions in the ESI‐MS spectra of Figure 1. The “naked” complexes observed include (Zn[**T**])_2_^4+^ and (Zn[**T**])_3_^6+^ (dimer and trimer at *m/z* 761 in Figures 1A and 2A), (Ni[**T**])_3_^6+^ (trimer at *m/z* 758 in Figures 1B and 2B), (Cd[**T**])_2_^4+^ (dimer at *m/z* 785 in Figures 1C and 2C), and (Fe[**T**])_3_^6+^ (trimer at *m/z* 757 in Figures 1D and 2D).

For architectural elucidation of the complexes formed from **T** and the four transition metals studied, the drift times of the separated ions (see t_D_ entries in Table S3, supporting information) were converted into experimental collision cross‐sections (Ω_exp_) through calibration of the drift time scale with ubiquitin calibrant ions (cf. Table S4 and Figure S13, supporting information); the resulting values are included in Table S3 (supporting information) and summarized for ready reference in Table 1. The Ω_exp_ values in Table 1 reveal how molecular size is affected by charge state and number of repeat units (dimer‐hexamer). As expected, adding a repeat unit, viz. a (Metal^2+^[**T**])(PF_6_)_2_ moiety, causes molecular expansion, whereas decreasing the charge state by adding a PF_6_ˉ counterion generally leads to a more compact structure due to the electrostatic attraction forces developing between the added anion and metal centers.

**Table 1:** Experimental collision cross‐sections (Ω_exp_) of the metallosupramolecular ions observed in the ESI‐IM‐MS mobilograms

		Ω_exp_ (Å^2^)^1^ of ([Metal^2+^{T}]_*n*_ [PF_6_]_2*n*–*x*_)^*x*+^				
Metal^2+^	Charge +*x*	Dimer *n* = 2	Trimer *n* = 3	Tetramer *n* = 4	Pentamer *n* = 5	Hexamer *n* = 6
Zn	+3	635				
	+4	663	861	927		
	+5		973			
	+6		1088			
	+7					1390
	+8					1434
	+9					1450
Ni	+3	637	705			
	+4		799	901		
	+5		990	1115		
	+6		1050	1128	1173, 1293	
	+7			1419	1162, 1354	1417, 1611^L^
	+8				1760^L^	1414
	+9				1769^L^	1457, 2127^L^
	+10					1519, 2142^L^
	+11					1813, 2138^L^
Cd	+3	637				
	+4	682	714	1150		
	+5		893	1154		
	+6			1107		
Fe	+3	640				
	+4		789			
	+5		967	1120	1168	
	+6		1024	1110	1129, 1251	1118
	+7			1388	1107, 1306	1379
	+8				1275, 1485, 1785^L^	1405
	+9				1424, 1733^L^	1399
	+10					1471

1 A value with a superscripted L denotes a linear architecture; all other values correspond to macrocyclic architectures.

The Ω_exp_ data in Table 1 confirm that the simplest mixture arises with Cd^2+^, which forms weak <tpy‐Cd^2+^‐tpy> bonds,^19^, ^45^ thereby allowing self‐assembly via reversible equilibria that yield the thermodynamically favored macrocyclic structures.^27^, ^46^ With a ligand exhibiting a 60^o^ dihedral angle between the two ligating sites (cf. Scheme 1), cyclic dimers, trimers, and tetramers appear to be the thermodynamically preferred products, as they can adopt geometries that readily accommodate a 60^o^ ligand.^25^, ^41^ Zn^2+^ forms stronger coordinative bonds,^19^, ^44^ but still permits reversible interconversions.^26^ In addition to cyclic dimers‐tetramers, this metal cogenerated cyclic hexamers, which had been observed in low yield with other 60^o^ ligands^25^ (a 120^o^ dihedral angle favors hexameric macrocycle formation^21^, ^22^). With Ni^2+^, which binds bis(terpyridine) ligands more strongly than Zn^2+^,^19^, ^44^ pentamers and hexamers with cyclic and linear architectures were coproduced with cyclic dimers‐tetramers; the linear structures are readily distinguished from the cyclic isomers by their markedly larger Ω_exp_ values (ΔΩ_exp_ ≈ 300–500 Å^2^). Finally, Fe^2+^, which gives rise to the most stable <tpy‐Metal^II^‐tpy> bonds among the four metals probed,^19^, ^44^ yielded cyclic dimers‐hexamers as well as a mixture of cyclic and linear pentamers. For the pentameric Ni^2+^ and Fe^2+^ complexes, macrocyclic conformers were observed in addition to linear and cyclic isomers. Such complexity corroborates that these strongly binding metals react under kinetic control, leading to kinetically trapped, less stable constructs.

The formation of both cyclic and linear hexamers (C_6_ and L_6_, respectively) during the self‐assembly of Ni^2+^ and ligand **T**, compared with the exclusive production of C_6_ complexes from Fe^2+^ and **T**, demonstrates how binding strength can affect ring formation. A linear isomer, like L_6_, would contain a tpy‐Metal^2+^ chain end with a partially complexed metal ion that may be stabilized by electrostatic interactions with nearby PF_6_ˉ anions. The higher strength of <tpy‐Metal^II^‐tpy> coordinative bonds for Fe^2+^ vis‐à‐vis Ni^2+^ (cf. Table S1, supporting information) prevents this from taking place with the former metal. Meanwhile, the higher thermodynamic stability of <tpy‐Metal^2+^‐tpy> bonds with Ni^2+^ and Fe^2+^, versus those with Cd^2+^ and Zn^2+^, is attested by the observation of Ni^2+^ and Fe^2+^ macrocycles in high charge states (10+ and 11+ for the hexamers), where significant like charge repulsion is present, but compensated for by the strength of the Ni^2+^‐**T** or Fe^2+^‐**T** coordinative bonds.

It is noteworthy that switching the metal exerts a minor effect on the same architecture. For example, the Ω_exp_ values of trimers with 5+ charges, viz. ([Metal^2+^{**T**}]_3_[PF_6_])^5+^, are 973, 990, 893, and 967 Å^2^ for Zn^2+^, Ni^2+^, Cd^2+^, and Fe^2+^, respectively; similarly, the Ω_exp_ values of macrocyclic hexamers with 8+ charges are 1434, 1414, and 1405 Å^2^ for Zn^2+^, Ni^2+^, and Fe^2+^, respectively (cf. Table 1). Computational predictions for the structures of the different *n*‐mers were therefore obtained only for Zn^2+^ (vide infra). For assessing the effect of *n*‐mer size on collision cross‐section, average Ω_exp_ values were calculated for all macrocyclic (C_*n*_) and linear (L_*n*_) species in Table 1. These are listed in Table 2, along with theoretical predictions (Ω_theo_) obtained by molecular modeling.

**Table 2:** Average Ω_exp_ and Ω_theo_ values of the metallosupramolecular ions comprising bis(terpyridine) ligand **T** and divalent transition metal^II^ ions (metal = Zn, Ni, Cd, Fe)

Architecture	Ω_exp_	(Å^2^)^1^	Ω_theo_	(Å^2^)^2^	Δ (%)^3^
Dimer, ([Metal^II^{**T**}]_2_[PF_6_]_4–*x*_}^*x*+^	C_2_	649 (9)	C_2_	694 (33)	6
			L_2_	735 (105)	
Trimer, ([Metal^II^{**T**}]_3_[PF_6_]_6–*x*_)^*x*+^	C_3_	904 (130)	C_3_	1057 (126)	15
Tetramer, ([Metal^II^{**T**}]_4_[PF_6_]_8–*x*_)^*x*+^	C_4_	1138 (157)	C_4_	1140 (132)	<1
Pentamer, ([Metal^II^{**T**}]_5_[PF_6_]_10–*x*_)^*x*+^	C_5_	1261 (119)	C_5_	1370 (163)	8
	L_5_	1762 (21.8)			
Hexamer, ([Metal^II^{**T**}]_6_[PF_6_]_12–*x*_)^*x*+^	C_6_	1484 (138)	C_6_	1512 (250)	2
	L_6_	2004 (262)			

1 Average Ω_exp_ values of all metals for each group of cyclic (C_*n*_) and linear (L_*n*_) species in Table 1; the numbers in parentheses are the corresponding standard deviations.

2 Average Ω_theo_ values from 50 candidate structures for each architecture; the metal atoms were parametrized for Zn(II); the numbers in parentheses are the corresponding standard deviations.

3 Difference in percentage between Ω_exp_ and Ω_theo_.

**Figure 4:**
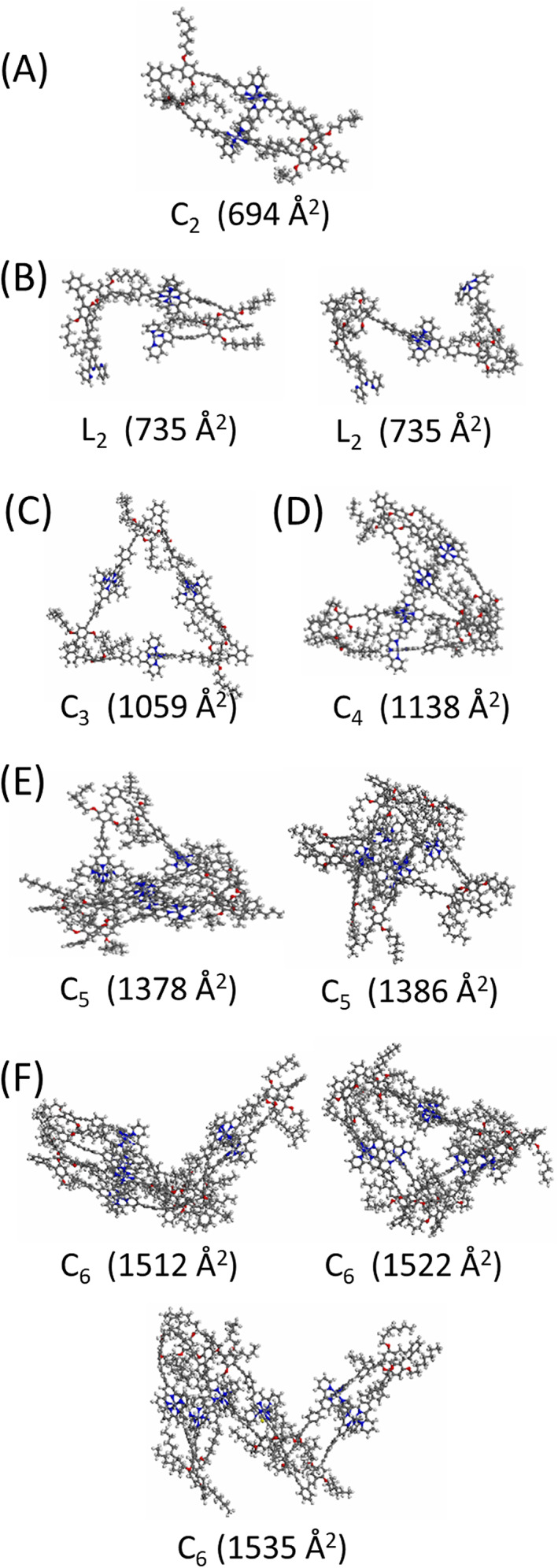
Simulated structures for Zn^2+^‐containing A, cyclic dimer C_2_, B, two linear dimers L_2_, C, cyclic trimer C_3_, D, cyclic tetramer C_4_, E, two cyclic pentamers (C_5_), and F, three cyclic hexamers. All structures are counterion‐free. The individual Ω_theo_ values are displayed under each structure

Figure 4 shows the computationally predicted structures for C_*n*_ macrocycles with Ω_theo_ values that are very close to the average calculated collision cross‐section of all 50 candidate structures generated for each *n*‐mer (cf. Table 2); the actual Ω_theo_ values of the depicted complexes are included in Figure 4. For the linear isomers, only dimer L_2_ was modeled, as the optimization of the large number of conformers possible with the longer chains was not tractable. Two stable conformations were found for L_2_, encompassing either a *cis* or a *trans* spiral arm positioning, but both had the same Ω_theo_ (735 Å^2^; cf. Figure 4). Such a spiral arm configuration would delay ring closure. The measured Ω_exp_ indicates, however, that only the cyclic dimer C_2_ is formed, presumably because the ends in this small *n*‐mer ultimately connect to form a macrocycle. The same scenario applies to the trimer and tetramer, which yield solely cyclic species. For the larger pentameric and hexameric complexes, however, the spiral geometry would increase the probability that loose ends remain uncoordinated, reconciling the observation of linear isomers.

Two structures having similar Ω_theo_ values but different numbers of protruding ligand vertices were obtained for the pentameric macrocycle C_5_; the conformer with a Ω_theo_ of 1378 Å^2^ carries one protruding vertex, while the structure with a Ω_Theo_ of 1386 Å^2^ comprises two exposed ligand vertices. Modeling of the hexameric macrocycles yielded three different conformations with similar Ω_theo_ values. As expected, the number of possible stable conformers increases with the number of repeat units.

The <tpy‐Metal^2+^‐tpy> coordination regions of the C_4_, C_5_, and C_6_ structures are in close proximity to one another. This is counterintuitive in view of the charge repulsion factors that exist in these structures, but the influence from ligand geometry overwhelms any repulsive forces and results in such “twisted” 3D structures that can accommodate the 60° dihedral angle in the ortho‐substituted bis (terpyridine) ligand **T**. Trimer C_3_, and to some extent dimer C_2_, accommodate this ligand geometry best, resulting in relatively “flat” 2D macrocyclic architectures (cf. Figure 4); in fact, it can be seen that the triangular geometry (which exhibits 60^o^ angles between the three sides) is retained in C_3_ without any major reorganization that the other macrocycles underwent.

## 4 CONCLUSIONS

Cyclic and linear complexes with the stoichiometry (Metal^2+^[**T**])_*n*_ (PF_6_)_2*n*_ (*n* = 2 to 6) were prepared by the self‐assembly of the 60°‐bis(terpyridine) ligand **T** with four different divalent transition metals (Zn, Ni, Cd, and Fe). ESI of these coordinatively bound complexes gave rise to a distribution of ([Metal^2+^{**T**}]_*n*_ [PF_6_]_2*n*–*x*_)^*x*+^ ions, ranging from *m/z* 750 to 2500 and having *x* = 2 to 11 charges, which were separated and thoroughly characterized using ESI‐MS and ESI‐IM‐MS. Due to the lability of the metal–ligand bonding between ligand **T** and Zn^2+^ or Cd^2+^, macrocyclization is favored with these transition metals because equilibration into thermodynamically favorable rings is allowed. In sharp contrast, Ni^2+^ and Fe^2+^ ions form strong metal–ligand bonds, thereby hindering equilibration and yielding kinetically stable complexes that include a larger variety of macrocycles than Zn^2+^ and Cd^2+^ ions, as well as linear pentameric and hexameric congeners. Overall, mixtures of dimeric, trimeric, tetrameric, pentameric, and hexameric complexes could be resolved and elucidated using ESI‐IM‐MS analysis. These data were supplemented by SEC separation and detection of macrocycles as well as ^1^H NMR analysis, both of which supported the ESI‐IM‐MS findings, but were unable to detect all constituents of the complex mixtures synthesized. Compositional identification and structural assignments were achieved through an analysis of isotope patterns following IM separation. The size, shape, and architecture of the detectable ions were determined from the Ω values deduced via the IM dimension, which ranged from approximately 600 to 2200 Å^2^. The magnitude of these Ω values was found to vary, depending on the degree of polymerization, structural stability, and charge state of the self‐assembled architectures. The experimental Ω values obtained via IM‐MS agreed well with theoretical predictions calculated using the Materials Studio and MOBCAL programs for geometry optimization and cross‐sectional derivation, respectively.

## Supporting information


**Figure S1.** Stacked ^1^H NMR (300 MHz) spectra of **T** in CDCl_3_ and (Fe[**T**][PF_6_]_2_)_*n*_, (Cd[**T**][PF_6_]_2_)_*n*_, and (Zn[**T**][PF_6_]_2_)_n_ in CD_3_CN (all isolated from the reaction solution). The 6,6″‐protons (those in ortho position to the N atoms) are shifted upfield from ca 8.7 ppm (non‐coordinated ligand **T**) to 7.2, 8.1, and 7.9 ppm for the Fe^2+^, Cd^2+^, and Zn^2+^ complexes, respectively.
**Figure S2.** Stacked ^1^H NMR (300 MHz, aromatic region) spectra of (Fe[**T**][PF_6_]_2_)_*n*_ (precipitate) and (Fe[**T**][PF_6_]_2_)_*n*_ (isolated from solution) in CD_3_CN.
**Figure S3.** ^1^H NMR (300 MHz, CD_3_CN) spectrum of (Fe[**T**][PF_6_]_2_)_*n*_ (purified precipitate from the reaction mixture).
**Figure S4.** ^1^H,^1^H COSY NMR (300 MHz, CD_3_CN) spectrum of (Fe[**T**][PF_6_]_2_)_*n*_ (purified precipitate from the reaction mixture). The green and blue squares mark cross peaks.
**Figure S5.** ^1^H NMR (300 MHz, CD_3_CN) spectrum of (Fe[**T**][PF_6_]_2_)_*n*_ (purified filtrate from the reaction mixture).
**Figure S6.** ^1^H NMR (300 MHz, CD_3_CN) spectrum of (Cd[**T**][PF_6_]_2_)_*n*_.
**Figure S7.** ^1^H,^1^H COSY NMR (300 MHz, CD_3_CN) spectrum of (Cd[**T**][PF_6_]_2_)_*n*_. The green, blue and red squares mark cross peaks.
**Figure S8.** ^1^H NMR (300 MHz, CD_3_CN) spectrum of (Zn[**T**][PF_6_]_2_)_*n*_.
**Figure S9.** ^1^H,^1^H COSY NMR (300 MHz, CD_3_CN) spectrum of (Zn[**T**][PF_6_]_2_)_*n*_. The green, blue and red squares mark cross peaks.
**Figure S10.** SEC contour plot (PDA detector) of (Fe[**T**][PF_6_]_2_)_*n*_ (purified precipitate from the reaction mixture) with the SEC trace at 581 nm and the UV–Vis absorption spectrum at 18.1 min retention time.
**Figure S11.** SEC contour plot (PDA detector) of (Fe[**T**][PF_6_]_2_)_*n*_ (purified filtrate from the reaction mixture) with the SEC trace at 581 nm and the UV–vis absorption spectrum at 18.1 min retention time.
**Figure S12.** SEC contour plot (PDA detector) of (Ni[**T**][PF_6_]_2_)_*n*_ with the SEC trace at 410 nm and the UV–vis absorption spectrum at 18.1 min retention time.
**Table S1.** Stability constants of Metal^2+^‐tpy complexes with first row transition metal ions.
**Table S2.** Calculated *m/z* values of the different charge states that can be formed upon ESI‐MS of the (Zn[**T**])_*n*_ (PF_6_)_2*n*_, (Ni[**T**])_*n*_ (PF_6_)_*2n*_, (Cd[**T**])_*n*_ (PF_6_)_*2n*_, and (Ni[**T**])_*n*_ (PF_6_)_*2n*_ macrocycles; bis (terpyridine ligand **T** has the elemental composition C_100_H_108_N_6_O_4_. Monoisotopic values are given for both the masses of the neutral complexes and the *m/z* values of the different charge states. Isobaric ions are shown in the same color.
**Table S3.** Mass‐to‐charge ratios (*m/z*), drift times (t_D_), and collision cross‐sections (Ω_exp_) of the ions observed in the ESI‐IM‐MS mobilograms of the complexes formed by coordinative self‐assembly of bis (terpyridine) ligand **T** with Zn^2+^, Ni^2+^, Cd^2+^, or Fe^2+^ ions.
**Table S4.** Drift times and corrected collision cross‐sections of ubiquitin calibrant ions.
**Figure S13.** Plot of corrected collision cross‐sections of the ubiquitin reference ions (Table S3) against the corresponding corrected drift times (deduced using the procedure described in ref. 4). The resulting calibration line was used to convert the measured drift times of the metallomacrocycles (and their linear isomers) into experimental collision cross‐sections.
