# Supplementary material for: Collision cross‐section analysis of self‐assembled metallomacrocycle isomers and isobars via ion mobility mass spectrometry
Source: Rapid Commun Mass Spectrom. 2020 Feb 8;34(Suppl 2):e8717. doi: 10.1002/rcm.8717 (PMC9285404; doi:10.1002/rcm.8717)
Supplement: Supplementary file 1 — Figure S1. Stacked 1H NMR (300 MHz) spectra of T in CDCl3 and (Fe[T][PF6]2) n , (Cd[T][PF6]2) n , and (Zn[T][PF6]2)n in CD3CN (all isolated from the reaction solution). The 6,6″‐protons (those in ortho position to the N atoms) are shifted upfield from ca 8.7 ppm (non‐coordinated ligand T) to 7.2, 8.1, and 7.9 ppm for the Fe2+, Cd2+, and Zn2+ complexes, respectively. Figure S2. Stacked 1H NMR (300 MHz, aromatic region) spectra of (Fe[T][PF6]2) n (precipitate) and (Fe[T][PF6]2) n (isolated from solution) in CD3CN. Figure S3. 1H NMR (300 MHz, CD3CN) spectrum of (Fe[T][PF6]2) n (purified precipitate from the reaction mixture). Figure S4. 1H,1H COSY NMR (300 MHz, CD3CN) spectrum of (Fe[T][PF6]2) n (purified precipitate from the reaction mixture). The green and blue squares mark cross peaks. Figure S5. 1H NMR (300 MHz, CD3CN) spectrum of (Fe[T][PF6]2) n (purified filtrate from the reaction mixture). Figure S6. 1H NMR (300 MHz, CD3CN) spectrum of (Cd[T][PF6]2) n . Figure S7. 1H,1H COSY NMR (300 MHz, CD3CN) spectrum of (Cd[T][PF6]2) n . The green, blue and red squares mark cross peaks. Figure S8. 1H NMR (300 MHz, CD3CN) spectrum of (Zn[T][PF6]2) n . Figure S9. 1H,1H COSY NMR (300 MHz, CD3CN) spectrum of (Zn[T][PF6]2) n . The green, blue and red squares mark cross peaks. Figure S10. SEC contour plot (PDA detector) of (Fe[T][PF6]2) n (purified precipitate from the reaction mixture) with the SEC trace at 581 nm and the UV–Vis absorption spectrum at 18.1 min retention time. Figure S11. SEC contour plot (PDA detector) of (Fe[T][PF6]2) n (purified filtrate from the reaction mixture) with the SEC trace at 581 nm and the UV–vis absorption spectrum at 18.1 min retention time. Figure S12. SEC contour plot (PDA detector) of (Ni[T][PF6]2) n with the SEC trace at 410 nm and the UV–vis absorption spectrum at 18.1 min retention time. Table S1. Stability constants of Metal2+‐tpy complexes with first row transition metal ions. Table S2. Calculated m/z values of the different charge states t [file RCM-34-0-s001.pdf]

Supporting Information

Collision cross-section analysis of self-assembled metallomacrocycle isomers and isobars via ion mobility mass spectrometry

Kevin J. Endres[1] | Kevin Barthelmes[2,3] | Andreas Winter[2,4] | Robert Antolovich[1] | Ulrich S. Schubert[2,4 *] | Chrys Wesdemiotis[1,5 *]

[1] Department of Polymer Science, The University of Akron, Akron, OH 44325, USA

[2] Laboratory of Organic and Macromolecular Chemistry (IOMC), Friedrich Schiller University Jena, Humboldtstr. 10, 07743 Jena, Germany

[3] Present address: Department of Materials and Applied Chemistry, Nihon University, 1-8-14 Kanda Surugadai, Chiyoda-ku Tokyo 101-8308, Japan

[4] Jena Center for Soft Matter (JCSM), Friedrich Schiller University Jena, Philosophenweg 7, 07743 Jena, Germany

[5] Department of Chemistry, The University of Akron, Akron, OH 44325, USA

***Correspondence**

Chrys Wesdemiotis (ORCID: 0000-0002-7916-4782), Department of Chemistry, The University of Akron, Akron, Ohio 44325, USA; Email: wesdemiotis@uakron.edu

Ulrich S. Schubert, Laboratory of Organic and Macromolecular Chemistry (IOMC), Friedrich Schiller Universität Jena, 07743 Jena, Germany; Email: Ulrich.Schubert@uni-jena.de

Table of Contents

Page(s)

1. Summary of NMR and SEC analysis of the metallomacrocycles S3-S4
2. NMR spectra of the metallomacrocycles S5-S10
3. SEC plots of the metallomacrocycles... S11-S12
4. Stabilities of $Metal^{2+}$-terpyridine coordinative bonds S13-S14
5. ESI-MS and ESI-IM-MS analysis S14-S20
    a. Isobaric species mass lists S14-S17
    b. Mass analysis and collision cross-sections....................................... S18-S19
    c. Calibration of drift time scale... S20
6. References S21

Experimental

Nuclear magnetic resonance (NMR) and size exclusion chromatography (SEC) analysis

NMR spectra were recorded on a 300 MHz NMR spectrometer (Bruker Fourier 300, Bruker BioSpin GmbH, Rheinstetten, Germany) in deuterated solvents at 300 K. Chemical shifts are reported in parts per million (ppm, δ scale) relative to the residual solvent signal. SEC analysis was performed on a Shimadzu system (Shimadzu Corp., Kyoto, Japan) equipped as follows: controller: SCL-10A VP; degasser: DGU-14A; pump: LC-10AD VP; auto sampler: SIL-10AD VP; oven: CTO-10A VP; UV-vis photo diode array (PDA) detector: SPD-10MA VP; RI detector: RID-10A; eluent: N,N-dimethylacetamide (DMAc) containing 0.08 wt-% ammonium hexafluorophosphate (NH_4PF_6); flow rate: 1 mL/min, temperature: 40 °C; column: Phenomenex (Torrance, CA, USA) Phenogel guard/10^5 Å/10^3 Å.

$(Fe[\mathbf{T}][PF_6]_2)_n$

The product partially precipitated from the reaction mixture. The purified filtrate and the precipitate were analyzed independently.

Precipitate from the reaction mixture
purple powder, 21% yield.
^{1}H NMR (300 MHz, CD_3CN) δ 9.20 (s, n·4H), 8.63 (d, J = 8.2 Hz, n·4H), 8.38 (d, J = 8.4 Hz, n·4H), 8.00-7.79 (m, n·10H), 7.70 (dd, J = 5.9, 3.4 Hz, n·2H), 7.46-7.31 (m, n·6H), 7.25-7.16 (m, n·6H), 7.15-7.05 (m, n·4H), 4.19 (t, J = 6.3 Hz, n·4H), 4.07 (t, J = 6.4 Hz, n·4H), 1.93-1.85 (m, n·4H), 1.82-1.70 (m, n·4H), 1.69-1.57 (m, n·4H), 1.54-1.19 (m, n·36H), 0.96-0.79 (m, n·12H).
SEC (DMAc + 0.08 wt-% NH_4PF_6) t = 18.13 min.

Sample isolated from the solution of the reaction mixture
purple powder, 40% yield.
^{1}H NMR (300 MHz, CD_3CN) δ 9.27-9.12 (m, n·4H), 8.63 (d, J = 8.1 Hz, n·4H), 8.38 (d, J = 8.3 Hz, n·4H), 8.02-7.78 (m, n·10H), 7.75-7.63 (m, n·2H), 7.48-7.29 (m, n·6H), 7.27-7.15 (m, n·6H), 7.15-7.04 (m, n·4H), 4.19 (t, J = 6.1 Hz, n·4H), 4.07 (t, J = 6.3 Hz, n·4H), 1.95-1.86 (m, n·4H), 1.83-1.70 (m, n·4H), 1.69-1.56 (m, n·4H), 1.54-1.19 (m, n·36H), 0.97-0.79 (m, n·12H).
SEC (DMAc + 0.08 wt-% NH_4PF_6) t = 18.12, 17.63 (shoulder), 17.15 min (shoulder).

$(Cd[\mathbf{T}][PF_6]_2)_n$

Orange powder, 63% yield.

^{1}H NMR (300 MHz, CD_3CN) δ 9.00-8.88 (m, n·4H), 8.77 (d, J = 8.2 Hz, n·4H), 8.29-8.15 (m, n·8H), 8.11 (d, J = 5.0 Hz, n·4H), 7.91-7.76 (m, n·6H), 7.67 (t, J = 5.9, 3.4 Hz, n·2H), 7.51 (dd, J = 7.4, 5.1 Hz, n·4H), 7.42-7.27 (m, n·6H), 7.16 (s, n·2H), 4.15 (t, J = 6.3 Hz, n·4H), 4.03 (t, J = 6.4 Hz, n·4H), 1.92-1.81 (m, n·4H), 1.80–1.67 (m, n·4H), 1.67-1.52 (m, n·4H), 1.51-1.15 (m, n·36H), 0.97-0.75 (m, n·12H).

SEC was attempted, but decomposition of the macrocycles on the column was observed.

$(Zn[\mathbf{T}][PF_6]_2)_n$

Orange powder, 36% yield.

^{1}H NMR (300 MHz, CD_3CN) δ 9.05-8.93 (m, n·4H), 8.73 (d, J = 8.1 Hz, n·4H), 8.25 (d, J = 8.1 Hz, n·4H), 8.22-8.14 (m, n·4H), 7.97-7.78 (m, n·10H), 7.74-7.61 (m, n·2H), 7.50-7.28 (m, n·10H), 7.18 (s, n·2H), 4.16 (t, J = 6.3 Hz, n·4H), 4.05 (t, J = 6.4 Hz, n·4H), 1.94-1.83 (m, n·4H) 1.81-1.68 (m, n·4H), 1.60 (d, J = 7.7 Hz, n·4H), 1.53-1.18 (m, n·36H), 0.99-0.76 (m, n·12H).

SEC was attempted, but decomposition of the macrocycles on the column was observed.

$(Ni[\mathbf{T}][PF_6]_2)_n$

Orange powder, 58% yield.

A ^{1}H NMR spectrum was not measured, because of the paramagnetic Ni(II) center.

SEC (DMAc + 0.08 wt-% NH_4PF_6) t = 18.09, 17.60 (shoulder), 17.01 min (shoulder).

NMR spectra of the metallomacrocycles

The ^{1}H NMR spectra (Figures S1-S9) display the metal-specific shifts of the terpyridine signals in the respective octahedral complexes: typically, the 6,6''-protons are shifted upfield from ca 8.7 ppm (non-coordinated ligand **T**) to 7.2, 8.1, and 7.9 ppm for the Fe^{2+}, Cd^{2+}, and Zn^{2+} complexes, respectively (cf. Figure S1). All spectra include signals of relatively sharp and distinct shape and reveal no residual free terpyridine signals. This indicates that closed well-defined structures were formed; in contrast, metallopolymers are known to exhibit very broad signals and residual non-coordinated terpyridine signals due to their end groups.[1,2] It is noteworthy that the ^{1}H NMR spectra of the purified precipitate of the Fe^{2+} complex and the sample isolated (and purified) from the reaction solution of (Fe[**T**])$_n$ (PF_6)$_{2n}$ look nearly identical (cf. Figure S2); however, the signals in the spectrum of the solution appear broadened, indicating a more uniform macrocyclization in the precipitate.

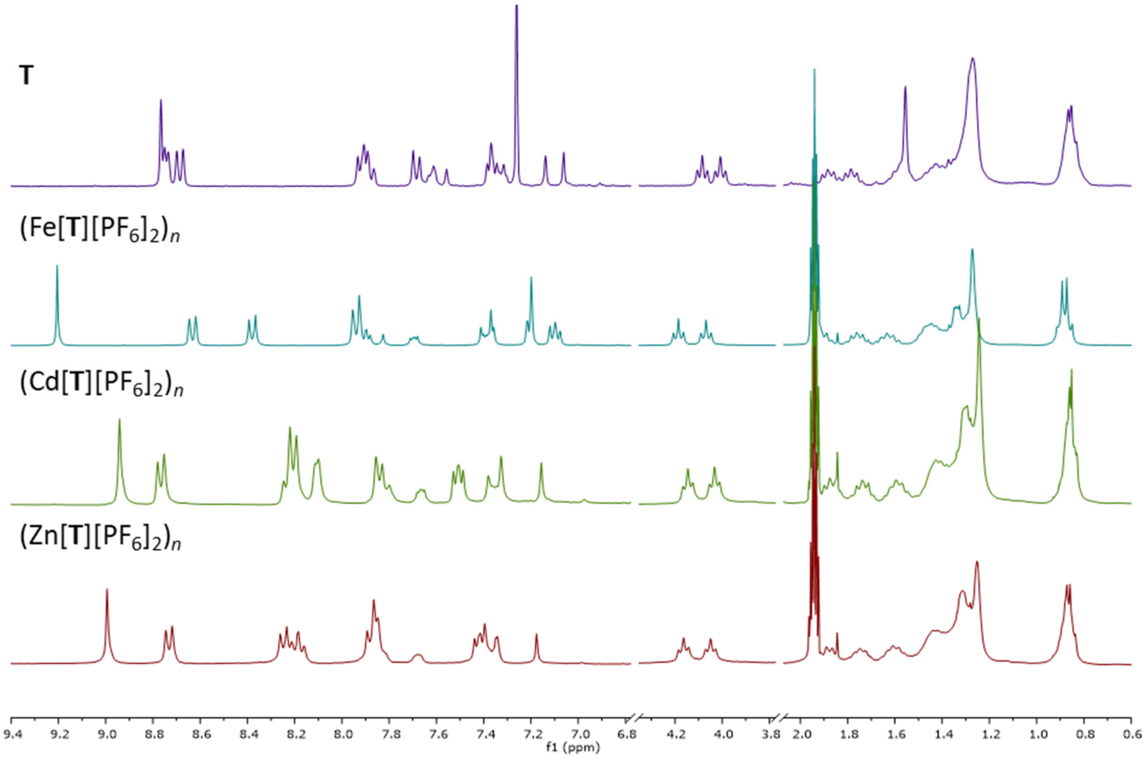

Figure S1. Stacked ^{1}H NMR (300 MHz) spectra of **T** in $CDCl_3$ and (Fe[**T**][PF_6]$_2$)$_n$, (Cd[**T**][PF_6]$_2$)$_n$, and (Zn[**T**][PF_6]$_2$)$_n$ in CD_3CN (all isolated from the reaction solution). The 6,6''-protons (those in ortho position to the N atoms) are shifted upfield from ca 8.7 ppm (non-coordinated ligand **T**) to 7.2, 8.1, and 7.9 ppm for the Fe^{2+}, Cd^{2+}, and Zn^{2+} complexes, respectively.

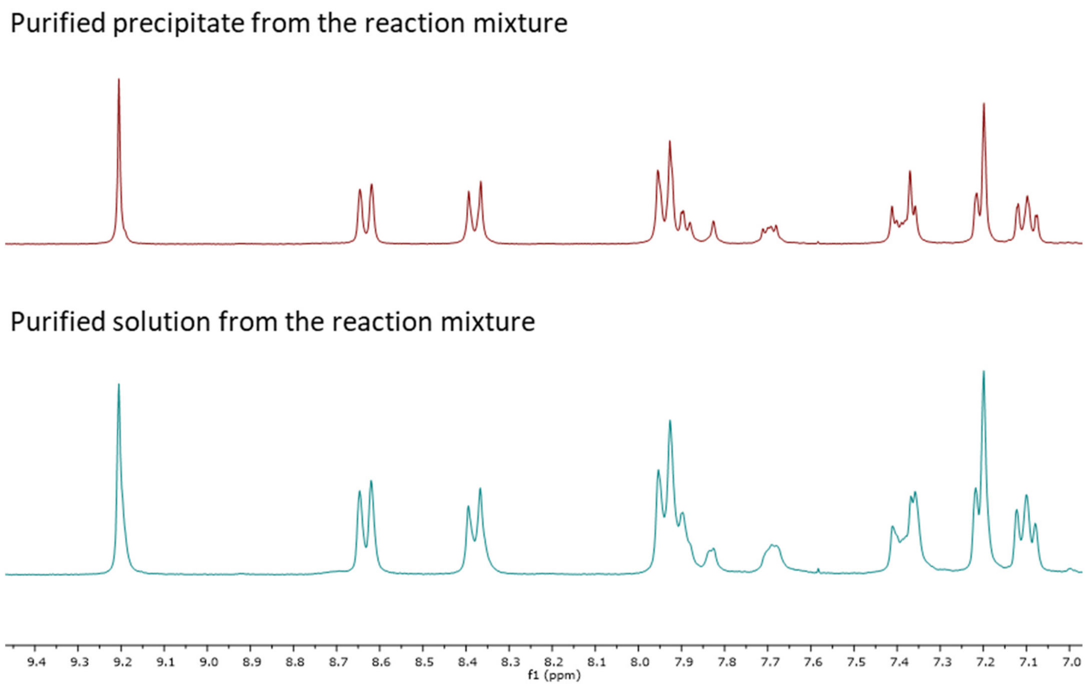

Figure S2. Stacked ^{1}H NMR (300 MHz, aromatic region) spectra of (Fe[**T**][PF_6]$_2$)$_n$ (precipitate) and (Fe[**T**][PF_6]$_2$)$_n$ (isolated from solution) in CD_3CN.

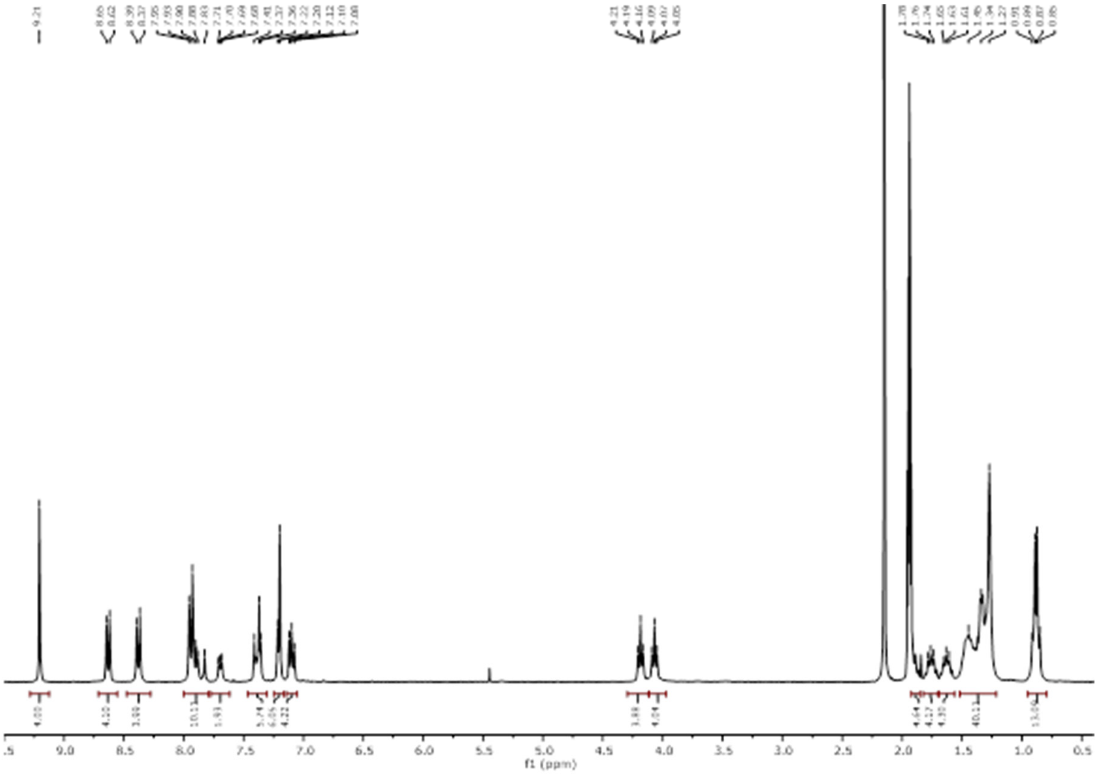

Figure S3. ^{1}H NMR (300 MHz, CD_3CN) spectrum of $(Fe[T][PF_6]_2)_n$ (purified precipitate from the reaction mixture).

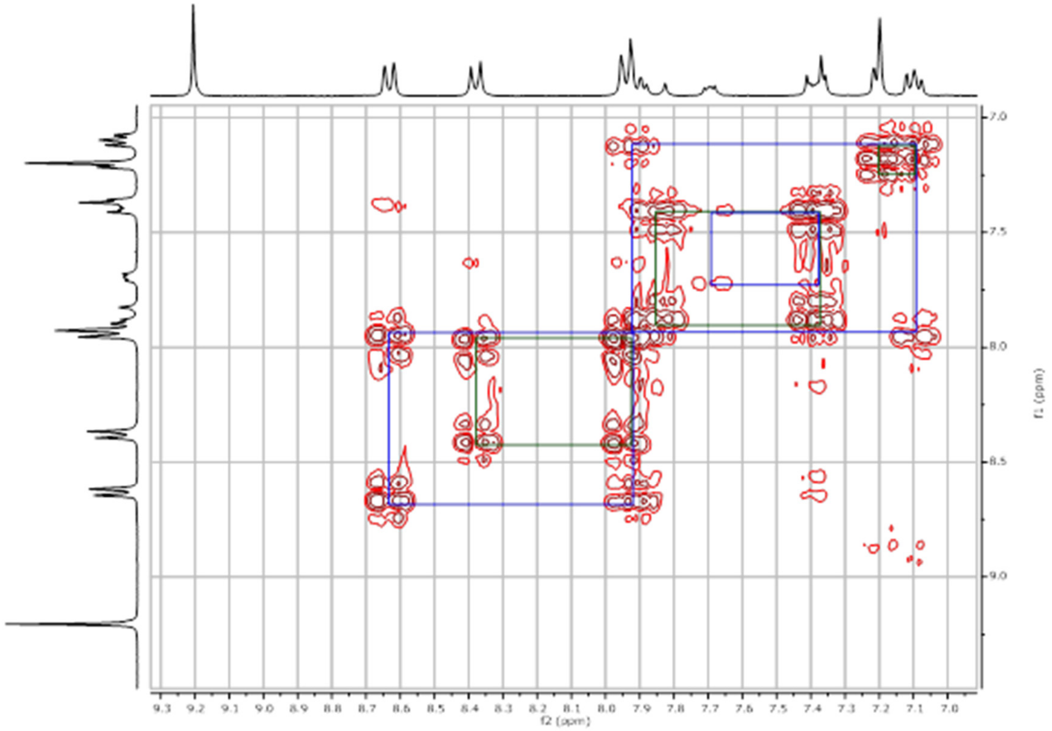

Figure S4. ^{1}H,^{1}H COSY NMR (300 MHz, CD_3CN) spectrum of $(Fe[\mathbf{T}][PF_6]_2)_n$ (purified precipitate from the reaction mixture). The green and blue squares mark cross peaks.

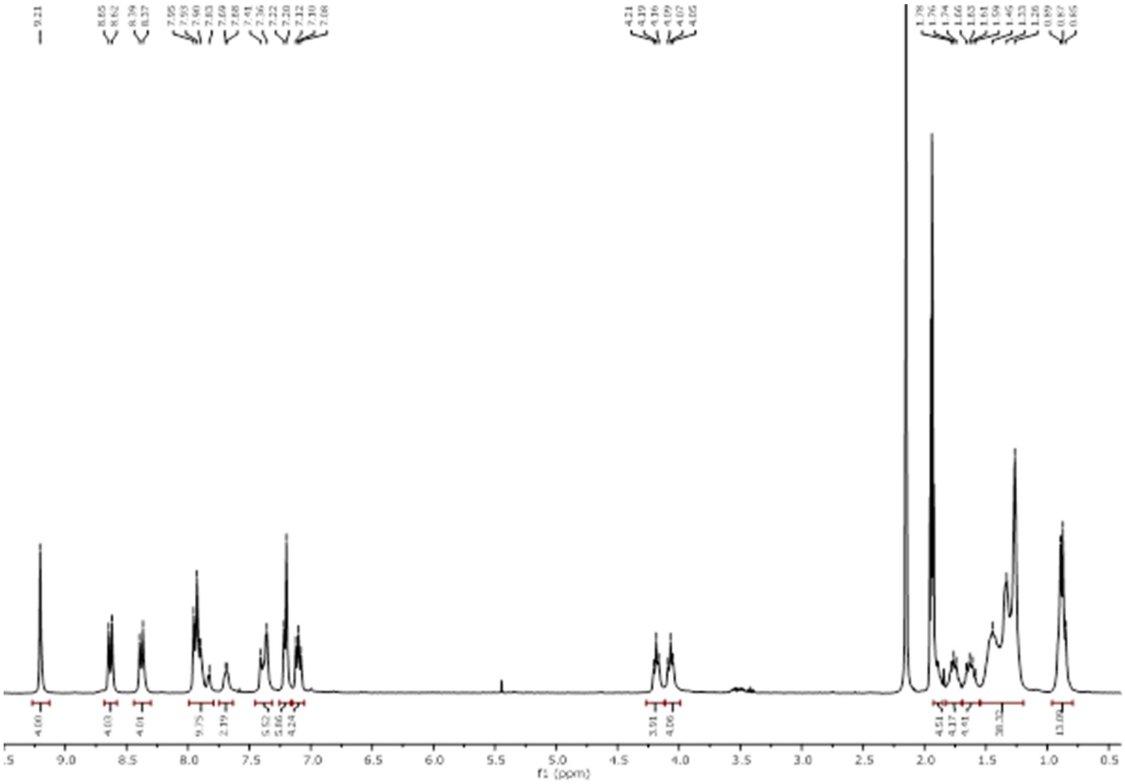

Figure S5. ^{1}H NMR (300 MHz, CD_3CN) spectrum of $(Fe[T][PF_6]_2)_n$ (purified filtrate from the reaction mixture).

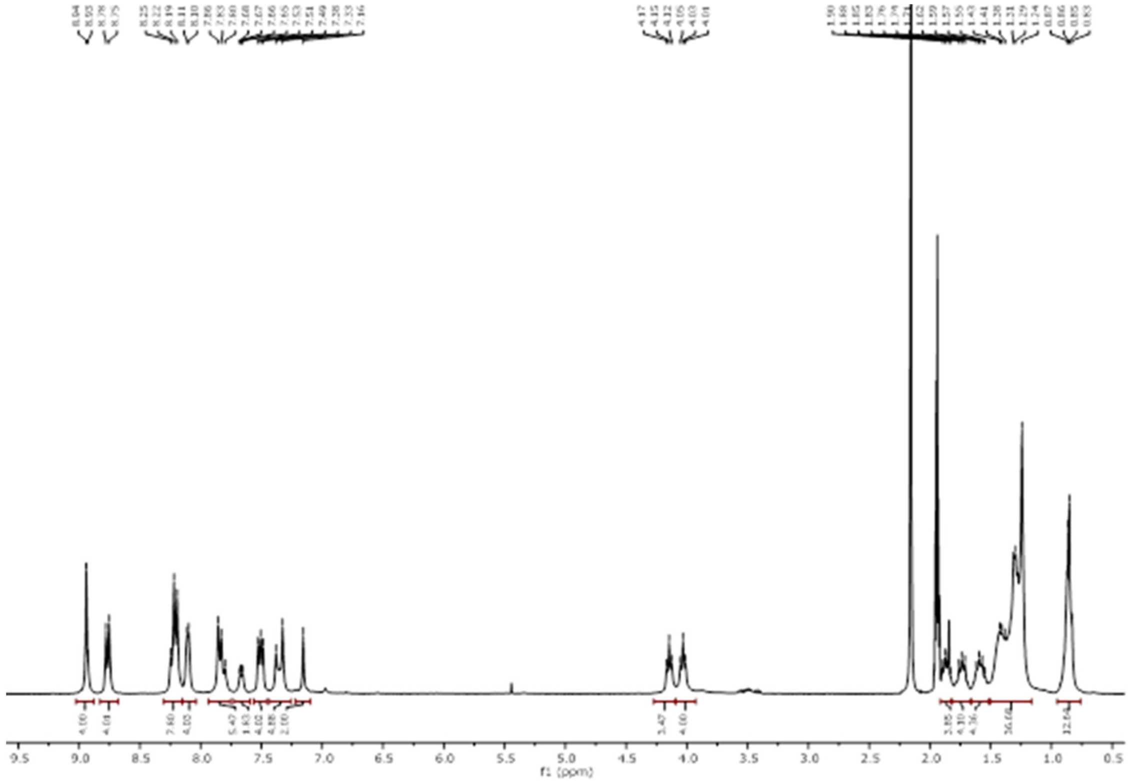

Figure S6. ^{1}H NMR (300 MHz, CD_3CN) spectrum of $(Cd[T][PF_6]_2)_n$.

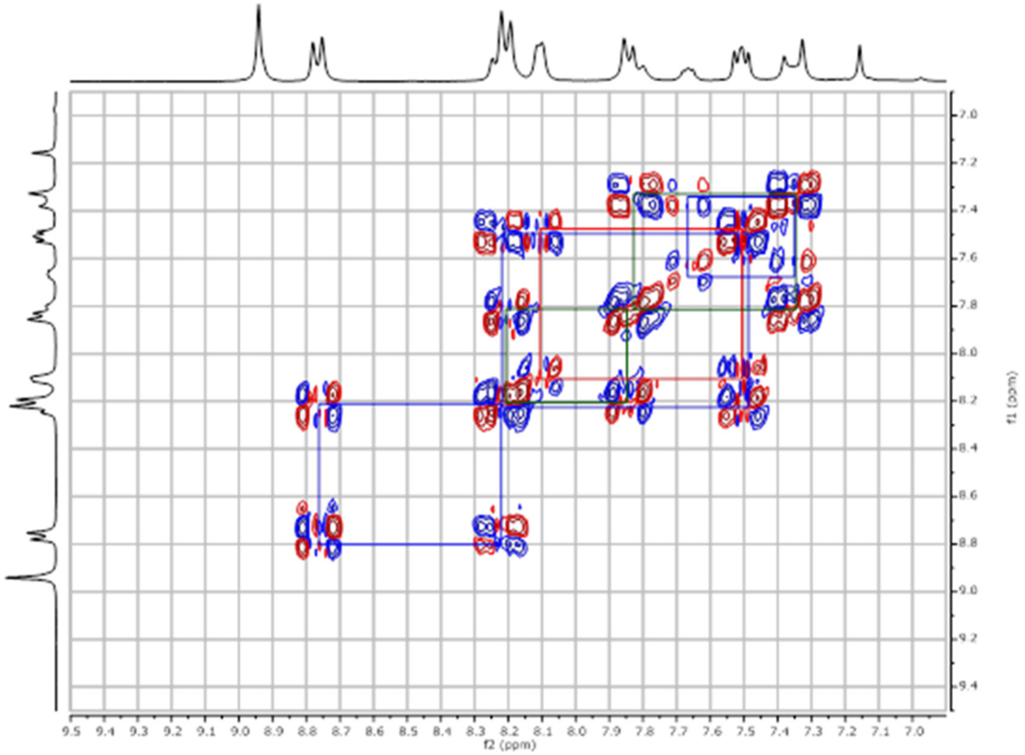

Figure S7. ^{1}H,^{1}H COSY NMR (300 MHz, CD_3CN) spectrum of $(Cd[\mathbf{T}][PF_6]_2)_n$. The green, blue, and red squares mark cross peaks.

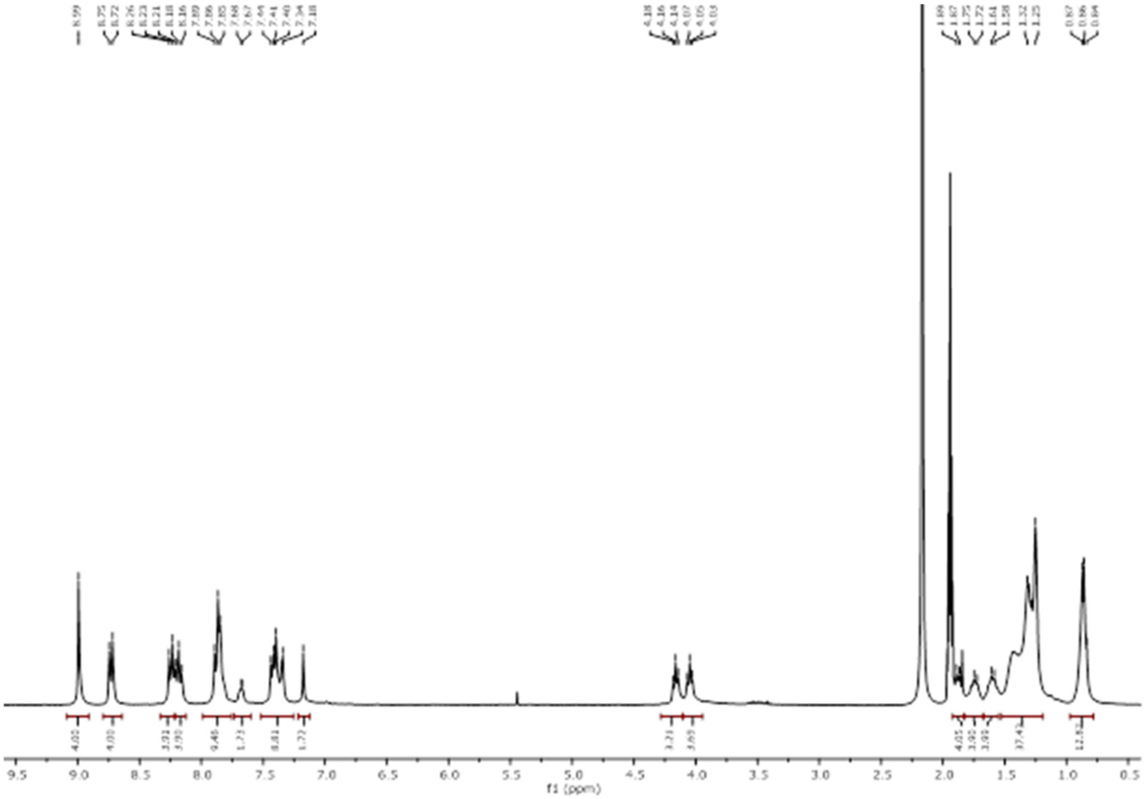

Figure S8. ^{1}H NMR (300 MHz, CD_3CN) spectrum of $(Zn[T][PF_6]_2)_n$.

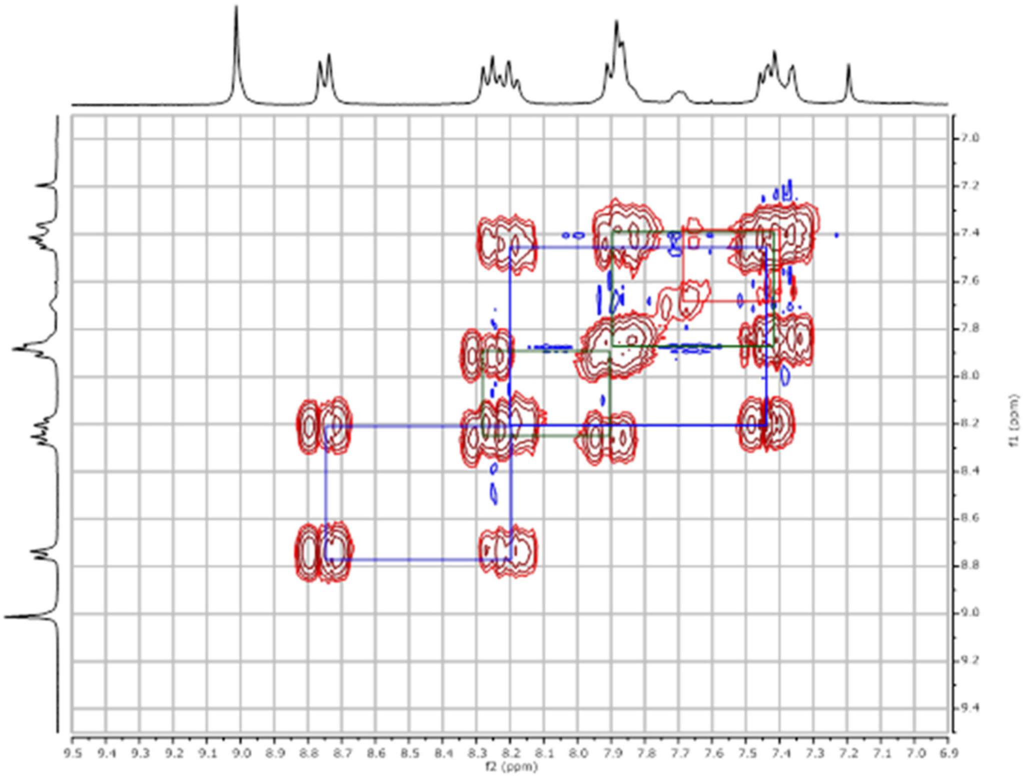

Figure S9. ^{1}H,^{1}H COSY NMR (300 MHz, CD_3CN) spectrum of $(Zn[\mathbf{T}][PF_6]_2)_n$. The green, blue, and red squares mark cross peaks.

SEC plots of the metallomacrocycles

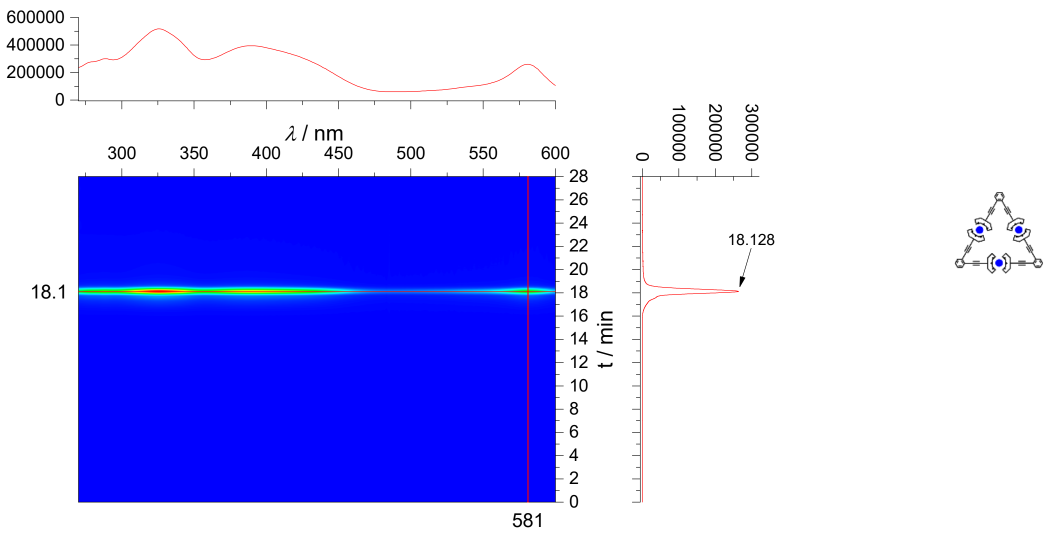

Figure S10. SEC contour plot (PDA detector) of (Fe[**T**][PF_6]$_2$)$_n$ (purified precipitate from the reaction mixture) with the SEC trace at 581 nm and the UV-vis absorption spectrum at 18.1 min retention time.

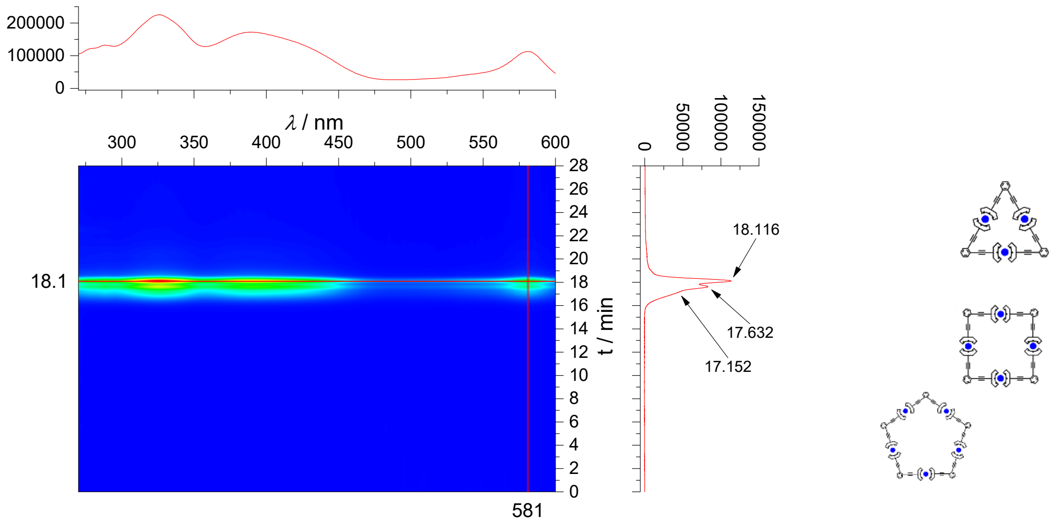

Figure S11. SEC contour plot (PDA detector) of (Fe[**T**][PF_6]$_2$)$_n$ (purified filtrate from the reaction mixture) with the SEC trace at 581 nm and the UV-vis absorption spectrum at 18.1 min retention time.

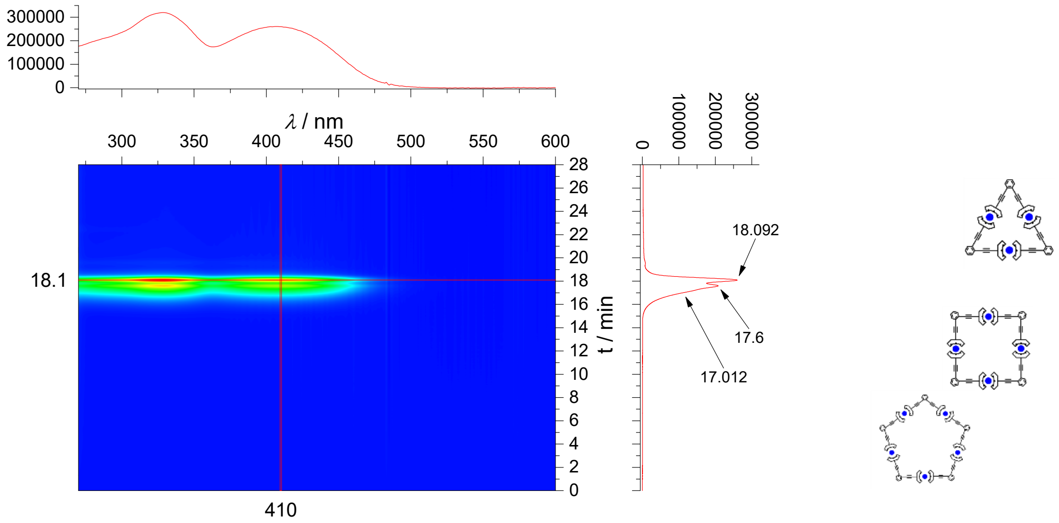

Figure S12. SEC contour plot (PDA detector) of (Ni[**T**][PF_6]$_2$)$_n$ with the SEC trace at 410 nm and the UV-vis absorption spectrum at 18.1 min retention time.

Stabilities of $Metal^{2+}$-terpyridine (tpy) complexes

Table S1. Stability constants (K) and bond enthalpies (ΔH^o) of $Metal^{2+}$-tpy complexes with first row transition metal ions.

$Metal^{2+}$	$\log K_1$ [a,b]	$\log K_2$ [a,c]	$\log \beta_2$ [d]	$-\Delta H^o_1$ [b,e] (kJ/mol)	$-\Delta H^o_2$ [c,e] (kJ/mol)
Cd^{2+}	5.1				
Zn^{2+}	6.7	5.2	11.9	61	61
Ni^{2+}	10.7	11.1	21.8	67	67
Fe^{2+}	7.1	13.8	20.9	80	80

[a] From the formation and dissociation rate constants in water using the stopped-flow method (for Cd^{2+}, Ni^{2+}, and Fe^{2+})[3] or via isothermal titration calorimetry in methanol (for Zn^{2+}).[4]

[b] For $Metal^{2+}$ + tpy $\rightleftharpoons$ $Metal^{2+}$(tpy).

[c] For $Metal^{2+}$(tpy) + tpy $\rightleftharpoons$ $Metal^{2+}(tpy)_2$.

[d] $\beta_2 = K_1K_2$, corresponding to $Metal^{2+}$ + 2tpy $\rightleftharpoons$ $Metal^{2+}(tpy)_2$.

[e] Via isothermal titration calorimetry in acetonitrile.[5]

The thermochemistry of $Metal^{2+}$-tpy bonds has been assessed by the stopped-flow method and by isothermal titration calorimetry.[3-5] The stability constants and bond enthalpies derived from these experiments for the metals investigated in our study are listed in Table S1.[3-5] These data point out that the stability of $Metal^{2+}$-tpy bonds in complexes with the <tpy-$Metal^{2+}$-tpy> connectivity increases in the order $Cd^{2+} < Zn^{2+} < Ni^{2+} < Fe^{2+}$. This order agrees well with a matrix-assisted laser desorption/ionization time-of-flight mass spectrometry (MALDI-TOF-MS) study, in which relative $Metal^{2+}$-tpy bond energies were estimated by comparing the laser intensities needed for the complete dissociation of $Metal^{2+}(L)_2$ complexes (L = 4'-(1,4,7-trioxa-octyl)-2,2':6',2"-terpyridine);[6] the laser intensity and, hence, binding strength followed the order $Cd^{2+} < Mn^{2+} < Cu^{2+} < Ni^{2+} < Fe^{2+} < Ru^{2+} < Co^{2+}$. NMR studies have further documented the occurrence of ligand exchange in Zn^{2+}-tpy complexes,[7] corroborating that complexation is reversible with metal ions forming weak coordinative bonds (i.e. Zn^{2+} and Cd^{2+}).

In the present study, $Metal^{2+}$-tpy complexes were characterized by ESI-MS and ESI-IM-MS. As a soft ionization method, ESI gently transfers intact molecular species from solution (where they are formed) into the mass spectrometer in the gas phase for identification

of their compositions and structures.[8] Thus, the complexes observed in our ESI-MS and ESI-IM-MS experiments must have been present in the solutions analyzed and reflect the solution chemistry taking place during their synthesis. For this reason, solution thermochemical data such as those presented above are essential for understanding the mixtures arising with different transition metal ions.

ESI-MS and ESI-IM-MS analysis of the metallomacrocycles

Table S2. Calculated *m/z* values of the different charge states that can be formed upon ESI-MS of the $(Zn[\mathbf{T}])_n (PF_6)_{2n}$, $(Ni[\mathbf{T}])_n (PF_6)_{2n}$, $(Cd[\mathbf{T}])_n (PF_6)_{2n}$, and $(Fe[\mathbf{T}])_n (PF_6)_{2n}$ macrocycles; bis(terpyridine ligand **T** has the elemental composition $C_{100}H_{108}N_6O_4$. Monoisotopic values are given for both the masses of the neutral complexes and the *m/z* values of the different charge states. Isobaric ions are shown in the same color.

Table S2.1. Zn metallomacrocycles

	Dimer	Trimer	Tetramer	Pentamer	Hexamer
n	2	3	4	5	6
Mass (Da)	3621.38	5432.07	7242.76	9053.45	10864.14
Charge			*m/z*		
1	**3476.42**	**5287.11**	7097.80	8908.49	10719.18
2	**1665.73**	**2571.08**	**3476.42**	4381.77	**5287.11**
3	**1062.17**	**1665.73**	2269.29	2872.86	**3476.42**
4	**760.39**	**1213.06**	**1665.73**	2118.40	**2571.08**
5		**941.45**	1303.59	**1665.73**	2027.87
6		**760.39**	**1062.17**	1363.95	**1665.73**
7			889.72	1148.39	1407.06
8			**760.39**	986.72	**1213.06**
9				860.98	**1062.17**
10				**760.39**	**941.45**
11					842.69
12					**760.39**

Table S2.2. Ni metallomacrocycles

	Dimer	Trimer	Tetramer	Pentamer	Hexamer
n	2	3	4	5	6
Mass (Da)	3609.40	5414.10	7218.80	9023.50	10828.20
Charge			*m/z*		
1	**3464.44**	**5269.14**	7073.84	8878.54	10683.24
2	**1659.74**	**2562.09**	**3464.44**	4366.79	**5269.14**
3	**1058.17**	**1659.74**	2261.31	2862.87	**3464.44**
4	**757.39**	**1208.57**	**1659.74**	2110.92	**2562.09**
5		**937.86**	1298.80	**1659.74**	2020.68
6		**757.39**	**1058.17**	1358.96	**1659.74**
7			886.30	1144.11	1401.93
8			**757.39**	982.98	**1208.57**
9				857.65	**1058.17**
10				**757.39**	**937.86**
11					839.42
12					**757.39**

Table S2.3. Cd metallomacrocycles

	Dimer	Trimer	Tetramer	Pentamer	Hexamer
n	2	3	4	5	6
Mass (Da)	3721.32	5581.98	7442.64	9303.30	11163.96
Charge			*m/z*		
1	**3576.36**	5437.02	7297.68	9158.34	11019.00
2	**1715.70**	**2646.03**	**3576.36**	4506.69	5437.02
3	**1095.48**	**1715.70**	2335.92	2956.14	**3576.36**
4	**785.37**	**1250.54**	**1715.70**	2180.87	**2646.03**
5		**971.44**	1343.57	**1715.70**	2087.83
6		**785.37**	**1095.48**	1405.59	**1715.70**
7			918.27	1184.08	1449.89
8			**785.37**	1017.95	**1250.54**
9				888.74	**1095.48**
10				**785.37**	**971.44**
11					869.95
12					**785.37**

Table S2.4. Fe metallomacrocycles

	Dimer	Trimer	Tetramer	Pentamer	Hexamer
n	2	3	4	5	6
Mass (Da)	3605.38	5408.07	7210.76	9013.45	10816.14
Charge	m/z				
1	**3460.42**	**5263.11**	7065.80	8868.49	10671.18
2	**1657.73**	**2559.08**	**3460.42**	4361.77	**5263.11**
3	**1056.83**	**1657.73**	2258.63	2859.52	**3460.42**
4	**756.39**	**1207.06**	**1657.73**	2108.40	**2559.08**
5		**936.65**	1297.19	**1657.73**	2018.27
6		**756.39**	**1056.83**	1357.28	**1657.73**
7			885.15	1142.68	1400.20
8			**756.39**	981.72	**1207.06**
9				856.53	**1056.83**
10				**756.39**	**936.65**
11					838.33
12					**756.39**

Table S3. Mass-to-charge ratios (*m/z*), drift times (t_D), and collision cross-sections (Ω_{exp}) of the ions observed in the ESI-IM-MS mobilograms of the complexes formed by coordinative self-assembly of bis(terpyridine) ligand **T** with Zn^{2+}, Ni^{2+}, Cd^{2+}, or Fe^{2+} ions.

Architecture	Metal (II)	Measured *m/z*	charge (+)	Theoretical *m/z*	Mass Error (ppm)	t_D (ms)[a]	Ω_{exp} (Å^2)[b]
Dimer	Zn	1063.833	3	1063.837	3.8	9.03	635 (1)
		761.377	4	761.387	13	4.69	663 (14)
	Ni	1059.167	3	1059.175	7.6	9.09	637 (1)
	Cd	1095.167	3	1095.155	11.0	9.09	637 (4)
		784.878	4	784.875	3.8	5.20	682 (12)
	Fe	1057.538	3	1057.509	27.4	9.21	640 (0)
Trimer	Zn	1214.813	4	1214.810	2.50	9.12	861 (11)
		942.857	5	942.857	0.0	7.31	973 (5)
		761.377	6	761.387	13.1	6.14	1088 (4)
	Ni	1661.419	3	1661.411	4.8	11.82	705 (2)
		1209.809	4	1209.818	7.4	7.82	799 (2)
		938.849	5	938.861	12.8	7.64	990 (3)
		758.057	6	758.042	19.8	5.57	1050 (4)
	Cd	1250.307	4	1250.295	9.6	5.87	714 (0)
		971.257	5	971.243	14.4	5.87	893 (0)
	Fe	1207.841	4	1207.818	19.0	7.58	789 (0)
		937.279	5	937.262	18.1	7.19	967 (5)
		756.567	6	756.557	13.2	5.23	1024 (0)
Tetramer	Zn	1668.232	4	1668.238	3.6	11.6	927 (7)
	Ni	1661.419	4	1661.411	4.8	10.7	901 (2)
		1300.186	5	1300.203	131	10.4	1115 (2)
		1059.167	6	1059.175	7.6	6.70	1128 (3)
		887.294	7	887.298	4.5	8.10	1419 (6)
	Cd	2335.612	3	2335.607	2.1	N/A	N/A
		1715.204	4	1715.214	5.8	11.2	1150 (2)
		1343.183	5	1343.179	3.0	7.10	1154 (0)
		1095.167	6	1095.155	11.0	4.30	1107 (0)
	Fe	1298.042	5	1298.004	29.2	10.5	1120 (0)
		1057.538	6	1057.509	27.4	6.40	1110 (4)
		885.734	7	885.727	7.9	7.70	1388 (6)
Pentamer	Zn	N/A	N/A	N/A	N/A	N/A	N/A
	Ni	1360.324	6	1360.293	22.8	7.40	1173 (3)
		1360.324	6	1360.293	22.8	9.50	1293 (3)
		1145.254	7	1145.256	1.7	4.90	1162 (5)
		1145.254	7	1145.256	1.7	7.20	1354 (0)
Linear		984.081	8	984.104	23.4	10.0	1760 (6)
Linear		858.540	9	858.541	1.2	7.52	1769 (5)
	Cd	N/A	N/A	N/A	N/A	N/A	N/A

	Fe	1658.764	5	1658.746	10.9	1167	1168 (2)
		1358.322	6	1358.295	19.9	6.70	1129 (3)
		1358.322	6	1358.295	19.9	8.80	1251 (0)
		1143.562	7	1143.543	16.7	4.30	1107 (0)
		1143.562	7	1143.543	16.6	6.59	1306 (0)
		982.495	8	982.480	153	4.40	1275 (10)
		982.495	8	982.480	15.3	6.50	1485 (0)
Linear		982.495	8	982.480	15.3	10.4	1785 (0)
		857.091	9	857.097	7.0	4.30	1424 (0)
Linear		857.091	9	857.097	7.0	7.10	1733 (0)
Hexamer	Zn	1409.223	7	1409.209	9.9	7.80	1390 (4)
		1214.813	8	1214.810	2.5	5.90	1434 (22)
		1063.833	9	1063.837	3.8	4.20	1430 (33)
Linear	Ni	1403.475	7	1403.501	18.5	11.3	1611 (3)
		1403.475	7	1403.501	18.5	8.10	1417 (0)
		1209.809	8	1209.818	7.4	5.08	1414 (5)
Linear		1059.167	9	1059.175	7.6	12.0	2127 (7)
		1059.167	9	1059.175	7.6	4.60	1457 (0)
Linear		938.849	10	938.861	12.8	9.40	2142 (5)
		938.849	10	938.861	12.8	3.90	1519 (8)
Linear		840.423	11	840.423	0.0	7.31	2138 (0)
		840.423	11	840.423	0.0	4.80	1813 (8)
	Cd	N/A	N/A	N/A	N/A	N/A	N/A
	Fe	1658.764	6	1658.746	10.9	6.60	1118 (0)
		1400.933	7	1400.930	2.1	7.60	1379 (0)
		1207.841	8	1207.818	19.0	5.70	1405 (5)
		1057.538	9	1057.509	27.4	4.20	1399 (0)
		937.279	10	937.262	18.1	3.60	1471 (0)

[a] Average values from triplicate measurements. The drift time distributions (cf. Figure 3b) were smoothed prior to t_D measurement.

[b] Standard deviations from the triplicate measurements are given in parentheses.

Table S4. Drift times and corrected collision cross-sections of ubiquitin calibrant ions.

z	MW	*m/z*	t_D (ms)[a]	Reported Ω (Å^2)[b]	Corrected Ωc (Å^2)[c]
4	8568.64	2142.16	12.21	972	1284
5	8569.65	1713.93	8.66	983	1039
9	8573.65	952.63	5.96	1670	980
10	8574.68	857.47	5.20	1730	914
11	8575.70	779.61	4.69	1800	865
12	8576.70	714.72	4.33	1890	832
13	8577.70	659.82	4.09	1980	805

[a] Measured in this study at a travelling wave velocity of 350 m/s and a travelling wave height of 7.5 V.

[b] From ref. 9.

[c] Corrected collision cross-sections, derived via the procedure described in ref. 10.

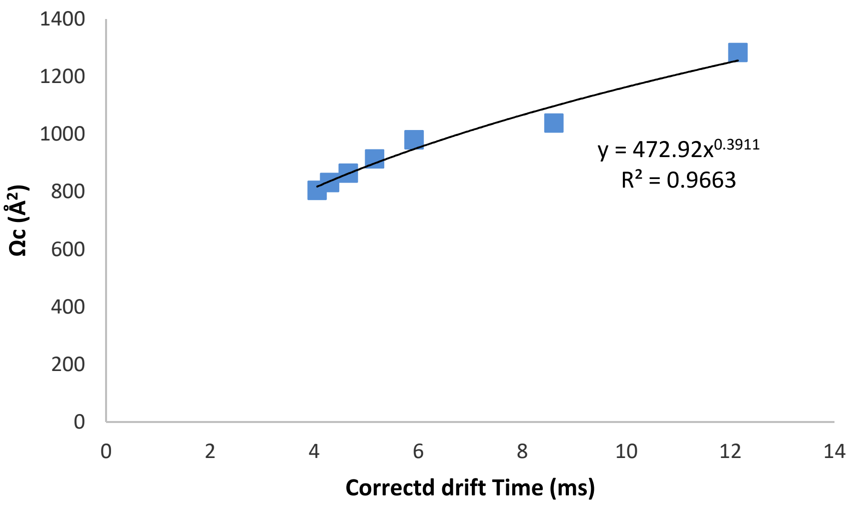

Figure S13. Plot of corrected collision cross-sections of the ubiquitin reference ions (Table S3) against the corresponding corrected drift times (deduced using the procedure described in ref. 10). The resulting calibration line was used to convert the measured drift times of the metallomacrocycles (and their linear isomers) into experimental collision cross-sections.

References

1. Schubert US, Winter A, Newkome GR. *Terpyridine-based Materials: For Catalytic, Optoelectronic and Life Science Applications.* Weinheim, Germany: Wiley-VCH Verlag & Co. KgaA; 2011.
2. Winter A, Friebe C, Hager MD, Schubert US. Synthesis of rigid π-conjugated mono-, bis-, tris-, and tetrakis(terpyridine)s: Influence of the degree and pattern of substitution on the photophysical properties. *Eur J Org Chem.* 2009;(6):801-809.
3. Holyer RH, Hubbard CD, Kettle SFA, Wilkins RG. The kinetics of replacement reactions of complexes of the transition metals with 2,2',2"-terpyridine. *Inorg Chem.* 1966;5(4):622-625.
4. Andres, P.R. Supramolecular assemblies and materials based on 2,2':6',2"-terpyridine metal complexes. *Ph.D. Dissertation*, TU Eindhoven, 2004 (DOI: 10.6100/IR578403).
5. Dobrawa R, Ballester P, Saha-Möller CR, Würthner F. Thermodynamics of 2,2':6',2"-terpyridine-metal ion complexation" In: Schubert US, Newkome GR, Manners I, eds. *Metal-Containing and Metallosupramolecular Polymers and Materials*. ACS Symposium Series, Washington, DC: ACS; 2006:43-62.
6. Meier MAR, Lohmeijer BGG, Schubert US. Relative binding strength of terpyridine model complexes under matrix-assisted laser desorption/ionization mass spectrometry conditions. *J Mass Spectrom.* 2003;38(5):510-516.
7. Dobrawa R, Würthner F. Metallosupramolecular approach toward functional coordination polymers. J Polym Sci Pol Chem. 2005;43(21):4981-4995.
8. Loo JA. Electrospray ionization mass spectrometry: a technology for studying noncovalent macromolecular complexes. *Int J Mass Spectrom.* 2000;200(1-3):175-186.
9. Fernandez-Lima FA, Blas RC, Russell DH. A study of ion-neutral collision cross-section values for low charge states of peptides, proteins, and peptide/protein complexes. *Int. Mass Spectrom.* 2010;298(1-3)111-118.
10. Thalassinos K, Grabenauer M, Slade SE, Hilton GR, Bowers MT, Scrivens JH. Characterization of phosphorylated peptides using traveling wave-based and drift cell ion mobility mass spectrometry. *Anal Chem.* 2009;81(1):248-254.
